# An immunomodulating peptide with potential to suppress tumour growth and autoimmunity

**DOI:** 10.1038/s41598-023-47229-y

**Published:** 2023-11-13

**Authors:** Michael Agrez, Christopher Chandler, Kristofer J. Thurecht, Nicholas L. Fletcher, Feifei Liu, Gayathri Subramaniam, Christopher B. Howard, Benjamin Blyth, Stephen Parker, Darryl Turner, Justyna Rzepecka, Gavin Knox, Anastasia Nika, Andrew M. Hall, Hayley Gooding, Laura Gallagher

**Affiliations:** 1 InterK Peptide Therapeutics Limited, New South Wales, Australia; 2 Auspep Pty Limited, Melbourne, Australia; 3 Centre for Advanced Imaging, University of Queensland, Brisbane, Australia; 4 Australian Institute for Bioengineering and Nanotechnology and the ARC Training Centre for Innovation in Biomedical Imaging Technologies, University of Queensland, Brisbane, Australia; 5 Department of Oncology,, Peter MacCallum Cancer Centre and Sir Peter MacCallum, University of Melbourne, Melbourne, Australia; 6 Concept Life Sciences, Edinburgh, Scotland

**Keywords:** Cytokines, Immunotherapy, Lung cancer, Autoimmunity, Lymphocytes

## Abstract

Cancers and autoimmune diseases commonly co-exist and immune checkpoint inhibitor therapy (ICI) exacerbates autoimmune pathologies. We recently described a lipidic peptide, designated IK14004, that promotes expansion of immunosuppressive T regulatory (Treg) cells and uncouples interleukin-2 from interferon-gamma production while activating CD8+ T cells. Herein, we report IK14004-mediated inhibition of Lewis lung cancer (LLC) growth and re-invigoration of splenocyte-derived exhausted CD4+ T cells. In human immune cells from healthy donors, IK14004 modulates expression of the T cell receptor α/β subunits, induces Type I IFN expression, stimulates natural killer (NK) cells to express NKG2D/NKp44 receptors and enhances K562 cytotoxicity. In both T and NK cells, IK14004 alters the IL-12 receptor β1/β2 chain ratio to favour IL-12p70 binding. Taken together, this novel peptide offers an opportunity to gain further insight into the complexity of ICI immunotherapy so that autoimmune responses may be minimised without promoting tumour evasion from the immune system.

## Introduction

Whilst targeting dysfunctional T cell mechanisms is an important approach in cancer immunotherapy, there remains a need to avoid indiscriminate autoimmune responses associated with treatment^1^. The incidence of autoimmune diseases is linked to cancer^2,3^ and anticancer therapy with immune checkpoint inhibitors (ICIs) has highlighted this further by exacerbating autoimmune pathologies^4–7^. On the other hand, ICIs act to limit tumour evasion from the immune system secondary to T cell exhaustion^8^. For example, Immune checkpoints such as the programmed cell death-1 (PD-1) receptor are major regulators of T cell exhaustion^9^ and in vitro PD-1 blockade has been shown to restore helper activity of exhausted CD4+ tumour-infiltrating lymphocytes (TILs) associated with enhanced IFN-γ production^10^.

The instructive cytokines, interleukin-2 (IL-2) and interleukin-12 (IL-12) activate cytotoxic CD8+ T cells and natural killer (NK) cells^11^. Sensing of IL-12 is mediated through the heterodimeric IL-12 receptor comprising IL-12Rβ1 and IL-12Rβ2 chains^12^. The IL-12Rβ2 chain is the signalling component of the IL-12 receptor and is critical for signal transduction downstream of the receptor complex^13–15^. IL-12 exists as two isoforms and much higher levels of IL-12p40 than IL-12p70 are normally expressed^16^. Moreover, IL-12p40 inhibits the effects of the IL-12p70 heterodimer^17^ by binding to the IL-12Rβ1 chain^18^. However, IL-12p70 can over-ride this inhibitory binding effect by signalling through IL-12Rβ2-mediated activation of the signal transducer and activator of transcription-4 (STAT4) which induces IL-12Rβ2 transcription^19,20^.

Dose-limiting toxicity due to IFN-γ overexpression has remained a concern with IL-12 therapy for cancer^21^. IL-12 is produced mainly by antigen-presenting cells and acts as a potent inducer of IFN-γ in T and NK cells that involves a feedback loop^22^ with induction of IL-12p40 mRNA by IFN-γ^23^. Modulation of IL-12Rβ2 receptor subunit expression is central to the regulation of IL-12 responsiveness^24^ and IL-12Rβ2 knock-out mice, which cannot utilise IL-12p70, develop lung cancer^25^. Moreover, the paradoxical effects of IL-12p70 in terms of controlling both autoimmune inflammation and enhancing CD8+ T cell-mediated cytotoxicity are well-recognised^26,27^. Not surprisingly, therefore, IL-12Rβ2 has been shown to offer protection against both spontaneous autoimmunity and malignancy in murine models^28^. Notably, in response to antigen and co-receptor stimulation, CD8+ T cells develop only weak effector functions in the absence of IL-12 or Type I interferons (IFN-α/β)^29^ and Type I IFNs can activate STAT4^30^. While STAT4 is widely recognised as a transcriptional activator and induces IFN-γ expression in NK cells, it can also function as a transcriptional repressor and how STAT4 activates one gene while repressing another within the same cell remains to be determined^31^.

The cytokine, IL-2, which is produced mainly by activated CD4+ T cells^32^, can rescue exhausted T cells^9^. IL-2 induces expression of the high-affinity IL-2 receptor subunit, IL-2Ra (CD25)^33^ and both IL-2 and CD25 are essential for differentiation of naïve CD8+ T cells into effector cytolytic T lymphocytes^34^. In T cells, transcription of CD25 is potently induced via either IL-2-mediated signalling or T cell receptor (TCR) activation^32^ and further enhanced upon phosphorylation of the T cell co-receptor, CD28^35,36^. The CD25 receptor subunit is absent or minimally expressed in resting T and NK cells^32^. However, in activated NK cells CD25 expression substantially increases their affinity for IL-2^37^ and is considered a marker of NK cell cytotoxicity^38^. In contrast to CD8+ T cells where IL-12Rβ2 expression is enhanced by either Type I IFNs or IFN-γ^39,40^, it is IL-2 that facilitates IL-12-mediated signalling in NK cells by upregulating expression of the IL-12 receptor^41,42^.

Activated human NK cells mediate lysis of tumour cells via natural cytotoxicity and antibody-dependent cellular cytotoxicity^43^. For example, IL-2 induces expression of the natural cytotoxic receptor (NCR), NKp44, that is only expressed on NK cells upon activation^44,45^. In addition, IL-2-mediated expression of the C-type lectin-like receptor, NKG2D, activates NK cells and co-stimulates CD8+ T cells^46^. NK cells can be primed by either IL-2 or by contact with a tumour cell that triggers tumour cell lysis^43^. Priming of NK cells can be achieved by IL-2 whereas “triggering” requires ligation of an additional activating receptor that can bind to a target ligand specific to stressed cells and which of the two is more effective is patient-dependent^47^. Importantly, IL-12-mediated signalling via the IL-12 receptor complex also upregulates NKG2D expression in both NK and CD8+ T cells^45^ and contributes to tumour cell lysis^48,49^.

The IL-12p70 heterodimer promotes induction of immunosuppressive CD4+ T regulatory (Treg) cells^27^ in contrast to their suppression by IL-12p40^50^. We have recently reported a novel lipidic peptide, designated IK14004, that induces expansion of Tregs, uncouples IL-2 from IFN-γ production and selectively increases IL-12p70 production by T cells over IL-12p40^51^. These findings have implications for regulating autoimmune responses. However, the peptide also activates CD8+ T cells and induces expression of NKG2D^51^. Given the dual role of IFN-γ in terms of promotion/prevention of tumour growth within the tumour micro-environment (TME)^52^, this raised the possibility of tumour inhibition by IK14004. Herein, we describe the effect of IK14004 in murine models of Lewis lung cancer (LLC) and on expression of activating receptors in immune cell subsets obtained from healthy human donors.

## Results

### IK14004 re-invigorates murine splenocyte-derived exhausted CD4+ T cells

**Figure 1:**
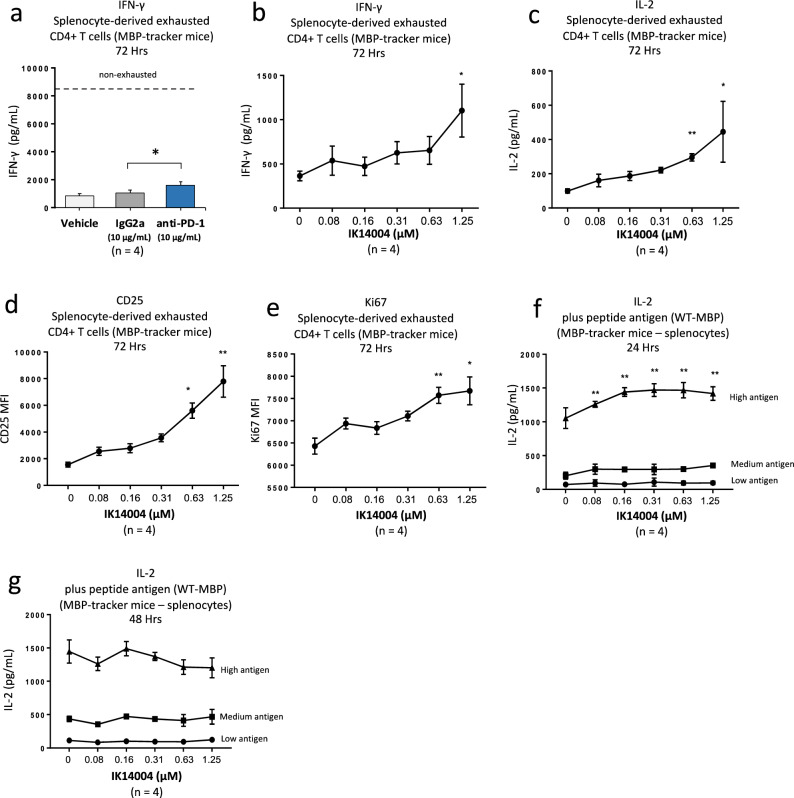
IK14004 re-invigorates transgenic splenocyte-derived exhausted CD4+ T cells. Splenocytes were derived from myelin basic protein (MBP)-tracker mice following ethics approval and rendered “exhausted” as described in the “Materials and methods” and in the “exhausted” model, isolated CD4+ T cells were re-stimulated with irradiated antigen-presenting cells, altered peptide ligand derived from MBP and either anti-PD-1 antibody, isotype control antibody or IK14004. In separate experiments naïve splenocytes derived from the MBP-tracker mouse model were exposed to Wild-Type-myelin basic protein (WT-MBP) at three different doses in in the absence presence of IK14004. Each tissue culture experiment was performed using triplicate wells (technical replicates) and cell-based flow cytometry/multiplex immunoassay experiments were repeated four times (n = experimental replicates) as indicated below each panel. All error bars represent standard error of the mean (SEM). In cell-based experiments with exhausted CD4+ T cells, the culture duration was 72 h, and experiments with non-exhausted naïve splenocytes the culture duration was either 24 or 48 h. Flow cytometry data are shown as mean fluorescence intensity (MFI). Dot plots and gating strategies are shown in Supplementary Fig. S1. (**a**) IFN-γ levels in supernatant from exhausted CD4+ T cell cultures in the presence of either anti-PD-1 mAb or IgG2a control antibody. Non-exhausted control cells are represented by the dotted line (Mean approximately 8000 pg/mL). (**b**) IFN-γ levels in supernatant from exhausted CD4+ T cell cultures in the presence of IK14004. (**c**) IL-2 levels from exhausted CD4+ T cell cultures in the presence of IK14004. (**d**) Expression of CD25 in exhausted CD4+ T cells. (**e**) Expression of Ki67 in exhausted CD4+ T cells. (**f**) IL-2 levels in supernatant from naïve splenocytes after 24 h**. **(**g**) IL-2 levels in supernatant from naïve splenocytes after 48 h. Data were analysed using a paired t test (**a**), non-parametric one-way ANOVA with Dunnett’s post-test comparing peptide with vehicle control (**b**–**e**) and two-way ANOVA with Dunnett’s post-test comparing groups at each dose level to vehicle (**f**, **g**). * P < 0.05, ** P < 0.01.

PD-1 blockade restores helper activity of exhausted CD4+ tumour-infiltrating lymphocytes (TILs) in vitro associated with enhanced IFN-γ production^10^. In preliminary studies we sought to validate the effect of anti-PD-1 antibody (10 µg/mL) in exhausted CD4+ T cells derived from myelin basic protein (MBP)-tracker mouse splenocytes (see “Materials and methods”). Non-exhausted control cells produce approximately 8000 pg/mL of IFN-γ compared with less than 1000 pg/mL from exhausted T cells (Fig. 1a). Re-stimulation of exhausted CD4+ T cells with irradiated antigen-presenting cells and a further dose of altered peptide ligand (APL) derived from MBP combined with anti-PD-1 nearly doubled the IFN-γ level within culture supernatant in comparison to the isotype control antibody, IgG2a, after 72 h (Fig. 1a). We then tested the effect of IK14004 in this model and in the presence of peptide, IFN-γ production increased significantly at the highest peptide concentration compared with vehicle control values (Fig. 1b). In addition, IL-2 production was enhanced (Fig. 1c), as was expression of CD25 (Fig. 1d**)** and the proliferation marker, Ki67 (Fig. 1e) after 72 h in culture.

We next tested the effect of IK14004 on the kinetics of IL-2 production upon TCR stimulation of CD4+ T cells using non-exhausted splenocytes derived from MBP-tracker mouse model. Naïve splenocytes were exposed to low (0.1 μM), medium (1 µM) or high (10 µM) concentrations of WT-MBP antigen and supernatants were collected 24 and 48 h later to assess IL-2 production. The addition of IK14004 induced an initial burst of IL-2 production in combination with the highest antigen dose after 24 h that plateaued at 160 nM (Fig. 1f) and this stimulatory effect was not maintained beyond 24 h (Fig. 1g). In contrast to the lack of effect of IK14004 on IL-2 production at 48 h, WT-MBP antigen alone at the higher doses (1 µM and 10 µM) further enhanced IL-2 levels after 48 h compared with the 24 h timepoint (Fig. 1f).

### IK14004 inhibits growth of murine Lewis lung cancer (LCC) and enhances IL-2/IL-12 receptor expression in splenocyte-derived T and NK cells

**Figure 2:**
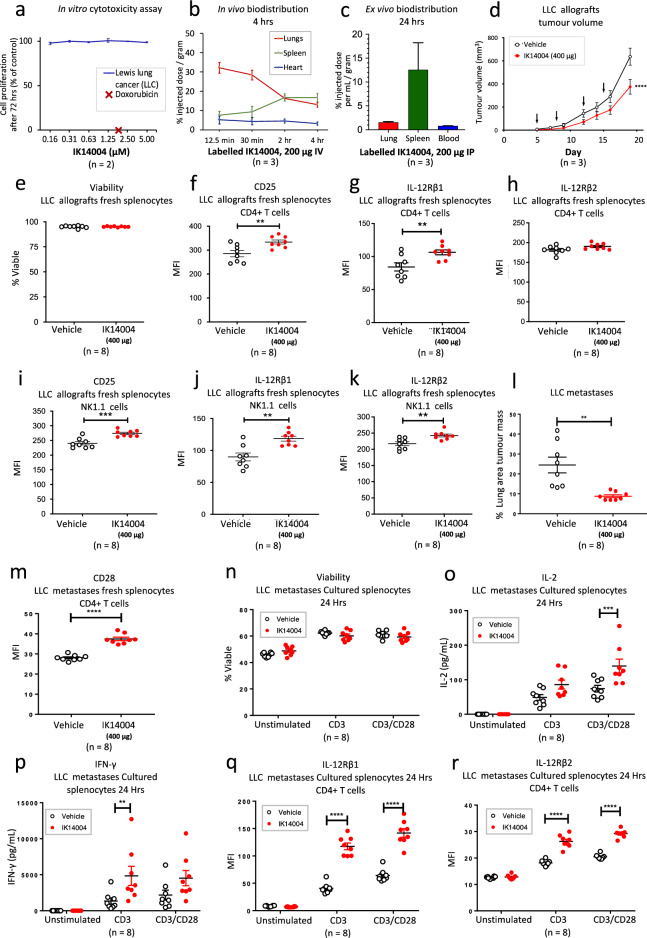
IK14004 inhibits growth of Lewis lung cancer (LCC) and enhances IL-2/IL-12 receptor expression in splenocyte-derived T and NK cells. Peptide biodistribution and tumour studies were undertaken with ethics approval and experiments to determine the effects of IK14004 in vitro*/*in vivo were performed as described in the “Materials and methods”. Five technical replicates per experiment were included in each of duplicate biological replicates to assess in vitro cytotoxicity of IK14004 and Doxorubicin. Biodistribution experiments were performed on 3 mice administered with ^64^NOTA-IK14004 and flow cytometry/ELISA experiments were performed with fresh splenocytes/supernatants from overnight splenocyte cultures, respectively. Two LLC models (subcutaneous allograft and lung metastasis model are identified below as AM, i.e., allograft model and LMM, i.e., lung metastasis model) were used to test effects of IK14004 administered I.P. twice per week for 2 weeks to groups of 8 mice (IK14004: 8 mice and vehicle control: 8 mice). Overnight splenocyte cultures were either left unstimulated or stimulated with anti-CD3 mAb alone or in combination with anti-CD28 mAb. All error bars represent standard error of the mean (SEM). Flow cytometry data are shown as percentage viability and mean fluorescence intensity (MFI). Dot plots and gating strategies are shown in Supplementary Figs. S2–S4. (**a**) Proliferation of LLC in vitro in the presence of IK14004 and Doxorubicin after 72 h. (**b**) Uptake of ^64^Cu-NOTA-conjugated IK14004 assessed in vivo within the lungs, spleen and heart (surrogate for blood) during the first 4 h. (**c**) Percentage retention of ^64^Cu-NOTA-conjugated IK14004 within the lungs, spleen and blood assessed ex vivo after 24 h. (**d**) LLC allograft growth after 2 weeks of treatment with IK14004. (**e**) Viability of freshly harvested splenocytes from the allografted mice (AM). (**f**) CD25 expression in CD4+ T cells within harvested splenocytes from the AM. (**g**) IL-12Rβ1 expression in CD4+ T cells within harvested splenocytes from the AM. (**h**) IL-12Rβ2 expression in CD4+ T cells within harvested splenocytes from the AM. (**i**) CD25 expression in NK cells within harvested splenocytes from the AM. (**j**) IL-12Rβ1 expression in NK cells within harvested splenocytes from the AM. (**k**) IL-12Rβ2 expression in NK cells within harvested splenocytes from the AM. (**l**) Percentage of lung area occupied by tumour in the LLC lung metastasis model (LMM). (**m**) CD28 expression in CD4+ T cells within harvested splenocytes in the LMM. (**n**) Viability of unstimulated and TCR-stimulated splenocytes following overnight culture in the LMM. (**o**) IL-2 in supernatants from unstimulated and TCR-stimulated splenocytes following overnight culture in the LMM. (**p**) IFN-γ in supernatants from unstimulated and TCR-stimulated splenocytes following overnight culture in the LMM. (**q**) IL-12Rβ1 expression in CD4+ T cells within unstimulated and TCR-stimulated splenocytes after overnight culture in the LMM. (**r**) IL-12Rβ2 expression in CD4+ T cells within unstimulated and TCR-stimulated splenocytes after overnight culture in the LMM. For tumour growth comparisons, data were analysed using an unpaired two-tailed t-test to assess differences between the two groups in both the allograft and the lung metastasis study. All other data were analysed using two-way ANOVA with Sidak’s multiple comparison. * P < 0.05, ** P < 0.01, *** P < 0.001, **** P < 0.0001.

Given in vitro immunomodulating effects previously reported for IK14004 (RSKAKNPLYR-4Adods; molecular weight 2021 Daltons) at mid-nanomolar/low micromolar concentrations^51^, we first assessed the effect of peptide on proliferation of the LLC cell line when cultured for 72 h and compared this with doxorubicin. No cytotoxic effect was observed in the presence of peptide whereas the positive control, doxorubicin, induced total cell kill at a concentration of 2.5 µM (Fig. 2a). In a preliminary in vivo peptide biodistribution study, ^64^Cu-labelled NOTA-conjugated IK14004 (200 µg) was administered via tail vein injection into Balb/C mice and marked accumulation of peptide was observed in the lung during the first 30 min (Fig. 2b). We next injected C57BL/6 mice intraperitoneally with the same dose to determine ex vivo peptide retention after 24 h. The percentage of injected dose remaining in the lung and spleen approximated 1.5% and 13% per gm, respectively (Fig. 2c). In blood, retention of IK14004 (Mol. Wt. 2021 Daltons) after 24 h was 0.75% per mL, i.e., approximately 0.75 μM, (Fig. 2c) which was within the effective dose range of peptide (0.31–1.25 µM) previously reported for human immune cells tested in vitro^51^. Hence, we selected a dose of 400 µg to inject intraperitoneally twice per week for two weeks into mice containing subcutaneous LLC allografts. Injections commenced on day 5 after tumour cell implantation and IK14004 significantly inhibited allograft growth at the termination of the experiment (Fig. 2d).

At the conclusion of the study, we sought to determine whether IK14004 treatment had affected expression of IL-2/IL-12 receptors in immune cell subsets within freshly harvested splenocytes. The viability of splenocytes harvested from vehicle- and peptide-treated mice approximated 100% (Fig. 2e). IK14004 induced a significant increase in expression of the high-affinity IL-2 receptor subunit, IL-2Rα (CD25), in CD4+ T cells compared with vehicle-treated mice (Fig. 2f) and enhanced expression of the IL-12Rβ1 receptor chain in CD4+ T cells (Fig. 2g) but not IL-12Rβ2 (Fig. 2h). In NK cells within the freshly harvested splenocyte populations, in vivo peptide treatment enhanced expression of CD25 (Fig. 2i), IL-12Rβ1 (Fig. 2j) and IL-12Rβ2 (Fig. 2k) compared with vehicle-treated mice.

To study the impact of peptide in a more challenging disease model, LLC cells were injected into the tail vein of C57BL/6 mice to assess tumour development in the lung. Five days after injection of LLC cells, IK14004 (400 μg) was administered intraperitoneally using the same treatment regime as in the subcutaneous allograft study. At the termination of the experiment, lung tumour growth was quantitated as the tumour area within serial lung sections stained with haematoxylin and eosin and the tumour burden was significantly suppressed in peptide-treated mice (Fig. 2l). In this model we assessed CD28 expression in CD4+ T cells within freshly harvested splenocytes and a marked increase in CD28 expression was observed in CD4+ T cells from IK14004-treated mice (Fig. 2m). We next sought to determine whether activating the TCR in CD4+/CD8+ T cells by overnight exposure of harvested splenocytes to anti-CD3/anti-CD28 monoclonal antibodies (mAbs) would affect production of IL-2/IFN-γ and expression of the IL-12 receptor chains, IL-12Rβ1/IL-12Rβ2. In a preliminary experiment, we compared the viability of cultured splenocytes isolated from peptide- and vehicle-treated mice and no differences were observed after 24 h (Fig. 2n). We then assessed IL-2 levels within culture supernatants and, in the absence of TCR stimulation, no IL-2 was detected after 24 h whereas significantly more IL-2 production was secreted by anti-CD3/anti-CD28-stimulated splenocytes obtained from peptide-treated mice compared with vehicle-treated mice (Fig. 2o) and similar effects were observed for IFN-γ production (Fig. 2p). Expression of IL-12Rβ1 in CD4+ T cells was also markedly increased in TCR-stimulated splenocyte populations obtained from peptide-treated mice compared with control mice (Fig. 2q) as was expression of IL-12Rβ2 (Fig. 2r).

### IK14004 enhances IL-2 production in human CD4+ T cells but not CD8+ T cells

**Figure 3:**
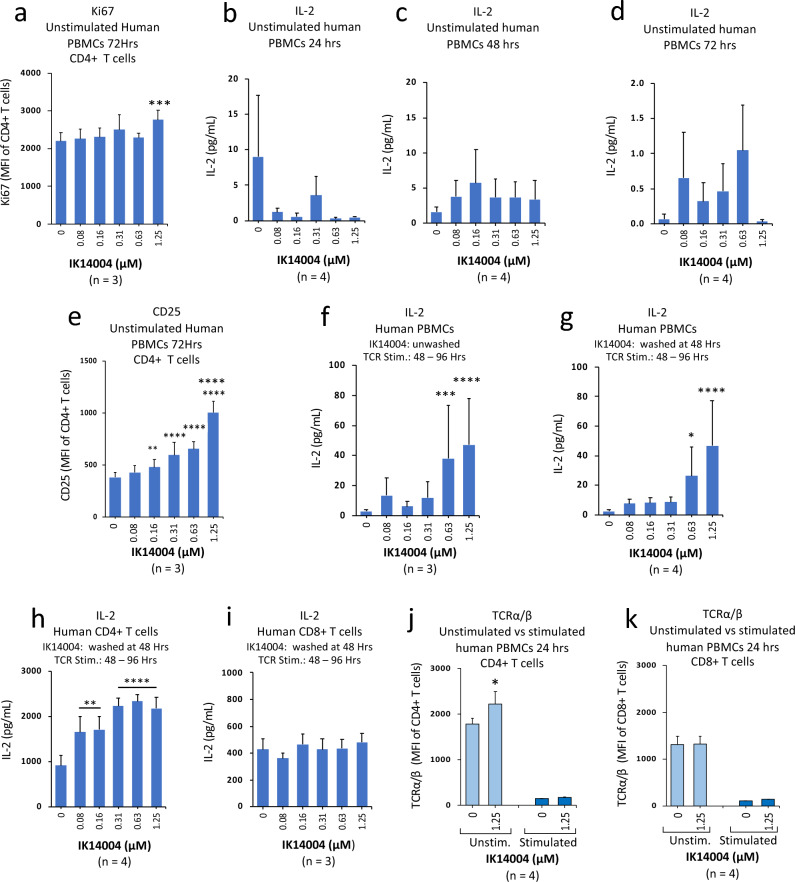
IK14004 enhances IL-2 production in human CD4+ T cells but not CD8+ T cells. Buffy coat samples were obtained from human volunteers following ethics approval. Non-TCR-stimulated PBMCs were cultured for 24–72 h. PBMCs were also exposed to IK14004 first for 48 h followed by either addition of anti-CD3 antibody for a further 48 h (unwashed cells; UC) or washed after the first 48 h to remove peptide followed by addition of anti-CD3 antibody for the remaining 48 h (washed cells; WC). Isolated CD4+ /CD8+ T cell cultures were exposed to peptide for the first 48 h followed by washing and then stimulated with anti-CD3/anti-CD28 antibodies for the next 48 h (washed T cells; WTC). Experiments to detect TCR α and β chain expression in CD4+ /CD8+ T cells within PBMC cultures were conducted with both unstimulated and anti-CD3-stimulated PBMCs over 24 h. Each tissue culture experiment was performed using triplicate wells (technical replicates) and cell-based flow cytometry/ELISA experiments were repeated at least three or four times (n = experimental replicates) as indicated below each panel. All error bars represent standard error of the mean (SEM). Flow cytometry data are shown as mean fluorescence intensity (MFI) and IL-2 production expressed as pg/mL. Dot plots and gating strategies are shown in Supplementary Figs. S5 and S6. (**a**) Expression of Ki67 in CD4+ T cells within unstimulated PBMC cultures. (**b**) IL-2 levels in supernatant from unstimulated PBMC cultures after 24 h. (**c**) IL-2 levels in supernatant from unstimulated PBMC cultures after 48 h. (**d**) IL-2 levels in supernatant from unstimulated PBMC cultures after 72 h. (**e**) Expression of CD25 in CD4+ T cells within unstimulated PBMC cultures after 72 h. (**f**) IL-2 levels in supernatant from unstimulated PBMC-UC cultures after 96 h. (**g**) IL-2 levels in supernatant from unstimulated PBMC-WC cultures after 96 h. (**h**) IL-2 levels in supernatant from isolated CD4+ T cell-WC cultures after 96 h. (**i**) IL-2 levels in supernatant from isolated CD8+ T cell-WC cultures after 96 h. (**j**) TCRα/β expression in CD4+ T cells within unstimulated versus TCR-stimulated PBMC cultures. (**k**) TCR α/β expression in CD8+ T cells within unstimulated versus TCR-stimulated PBMC cultures. Data were analysed by either two-way ANOVA with Dunnett’s post-test (**a**, **e**) or Sidak’s post-test (**f**, **g**, **h**, **i**), one-way ANOVA with Dunnett’s post-test (**b**, **c**, **d**) or two-way ANOVA with Holm-Sidak’s multiple comparisons post-test (**j**, **k**). * P < 0.05, ** P < 0.01, *** P < 0.001, **** P < 0.0001.

The cytokine, IL-2, is produced primarily by CD4+ T cells following antigen stimulation^32^ and in preliminary studies we examined the effect of IK14004 on proliferative capacity of CD4+ T cells within unstimulated PBMC cultures after 72 h. Only at the highest peptide concentration (1.25 µM) was Ki67 expression in CD4+ T cells significantly enhanced (Fig. 3a). IL-2 induces CD25 expression^33^ and we next sought to determine whether IK14004 induces either IL-2 production or CD25 expression in CD4+ T cells from non-TCR-stimulated PBMCs. In the absence of anti-CD3 mAb, exposure to IK14004 did not result in any dose-dependent increases in IL-2 levels within culture supernatants after either 24 h (Fig. 3b), 48 h (Fig. 3c) or 72 h (Fig. 3d). In contrast, at 72 h, expression of CD25 on CD4+ T cells exposed to peptide was significantly enhanced at all but the lowest concentration of IK14004 when compared to vehicle control (Fig. 3e).

Since IK4004 did not enhance IL-2 production by non-TCR-stimulated PBMCs after 48 h, we asked whether exposure of unstimulated T cells to peptide first for 48 h, followed by washing off peptide with fresh medium and then stimulating the TCR for the next 48 h, would lead to peptide-mediated IL-2 production. We first sought to examine IL-2 levels without the cell-washing step and IL-2 production was significantly enhanced at higher peptide concentrations (Fig. 3f). Inclusion of the washing step after the first 48 h to remove peptide followed by TCR stimulation resulted in nearly identical levels of IL-2 production 48 h later (Fig. 3g) which suggested that peptide removal had not removed any IL-2 possibly induced in response to peptide-pre-incubation alone. We next examined the effect of washing out peptide from isolated CD4+ /CD8+ T cell cultures after 48 h followed by TCR stimulation with anti-CD3/anti-CD28 mAbs for a further 48 h. Surprisingly, a dose-dependent increase in IL-2 production was only observed in CD4+ T cell cultures (Fig. 3h) but not from cultured CD8+ T cells (Fig. 3i). Taken together with no effect of peptide on IL-2 production in non-TCR-stimulated PBMC cultures after 48 h (Fig. 3c), this raised the possibility that IK14004 may prime the TCR differently in CD4+ as opposed to CD8+ T cells. The TCR consists of 2 subunits, α and β, and both chains are involved in TCR activation^53^. Hence, we selected the highest peptide concentration (1.25 µM) and compared its effect on TCRα/β expression in CD4+ /CD8+ T cells within unstimulated and stimulated PBMC cultures. Within non-TCR-stimulated PBMCs, exposure to IK14004 significantly enhanced expression of α/β subunits in CD4+ T cells after 24 h (Fig. 3j) whereas no effect was seen in CD8+ T cells (Fig. 3k). In TCR-activated PBMCs expression of the TCR subunits was barely detectable in either T cell subset probably due to epitope-masking by anti-CD3 mAb.

### IK14004 increases the IL-12RΒ2: IL-12RΒ1 expression ratio irrespective of TCR activation in CD4+ /CD8+ T cells

**Figure 4:**
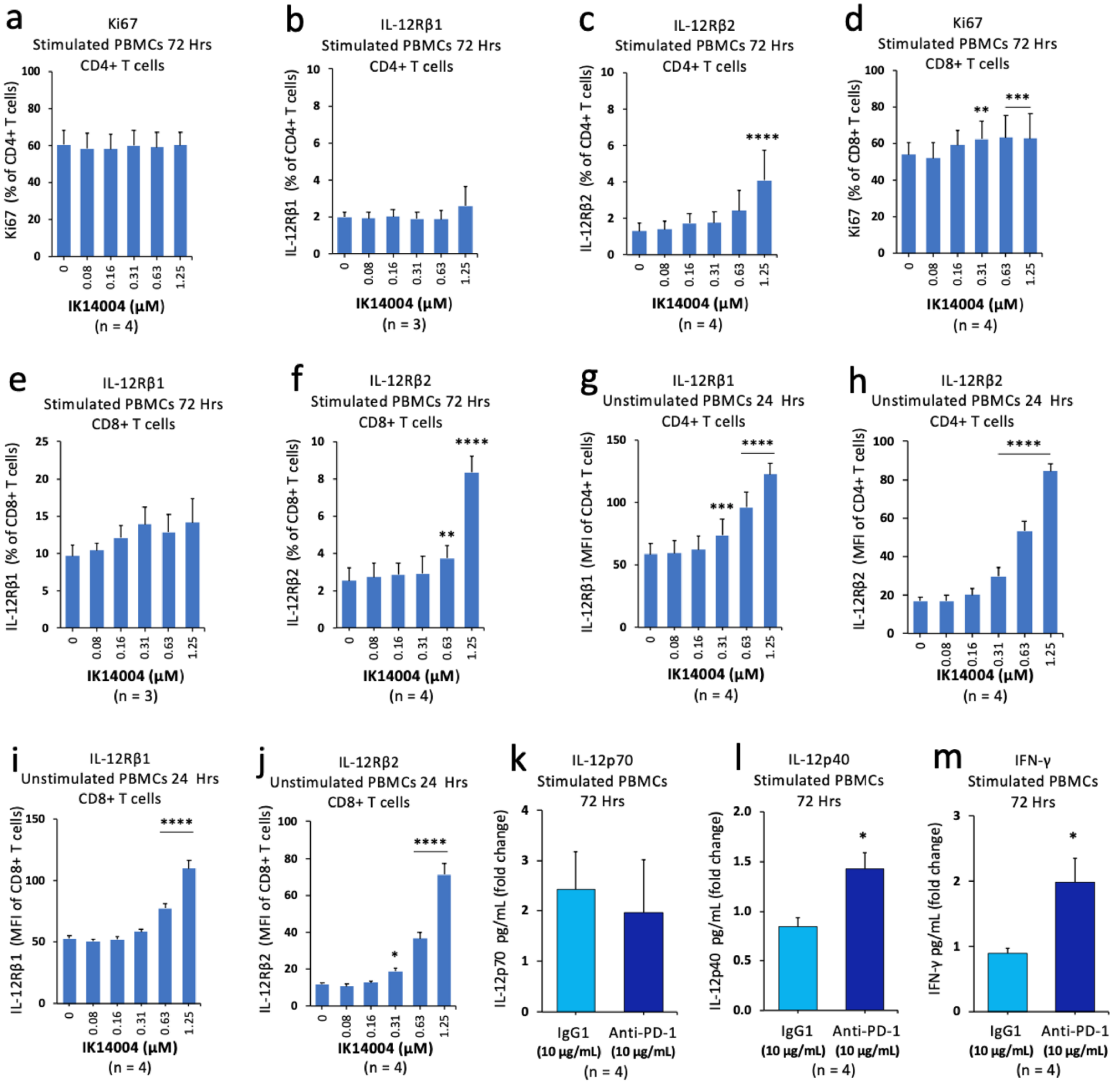
IK14004 increases the IL-12RΒ2: IL-12RΒ1 expression ratio irrespective of TCR activation in CD4+ /CD8+ T cells. Buffy coat samples were obtained from human volunteers following ethics approval. PBMC cultures were either not stimulated or stimulated with anti-CD3 antibody for either 24 or 72 h. Each tissue culture experiment was performed using triplicate wells (technical replicates) and cell-based flow cytometry experiments in the presence of IK14004 were repeated at least three or four times (n = experimental replicates) as indicated below each panel. ELISA studies were performed to detect IL-12p40/IL-12p70 and IFN-γ levels in supernatants from anti-CD3-stimulated PBMC cultures exposed to either anti-PD-1 mAb or isotype control IgG1 antibody. All error bars represent standard error of the mean (SEM). Flow cytometry data are shown as either mean fluorescence intensity (MFI) or percentage of receptor-expressing cells and dot plot/gating strategies are shown in Supplementary Figs. S7 and S8. (**a**) Percentage of Ki67-expressing CD4+ T cells within stimulated PBMC cultures. (**b**) Percentage of IL-12Rβ1-expressing CD4+ T cells within stimulated PBMC cultures. (**c**) Percentage of IL-12Rβ2-expressing CD4+ T cells within stimulated PBMC cultures. (**d**) Percentage of Ki67-expressing CD8+ T cells within stimulated PBMC cultures. (**e**) Percentage of IL-12Rβ1-expressing CD8+ T cells within stimulated PBMC cultures. (**f**) Percentage of IL-12Rβ2-expressing CD8+ T cells within stimulated PBMC cultures. (**g**) Expression of IL-12Rβ1 in CD4+ T cells within unstimulated PBMC cultures. (**h**) Expression of IL-12Rβ2 in CD4+ T cells within unstimulated PBMC cultures. (**i**) Expression of IL-12Rβ1 in CD8+ T cells within unstimulated PBMC cultures. (**j**) Expression of IL-12Rβ2 in CD8+ T cells within unstimulated PBMC cultures. (**k**) IL-12p70 levels in supernatant from stimulated PBMC cultures exposed to either anti-PD-1 mAb or isotype control IgG1 antibody. (**l**) IL-12p40 levels in supernatant from stimulated PBMC cultures exposed to either anti-PD-1 mAb or isotype control IgG1 antibody. (**m**) IFN-γ levels in supernatant from stimulated PBMC cultures exposed to either anti-PD-1 mAb or isotype control IgG1 antibody. Data were analysed by two-way ANOVA with Dunnett’s post-test for studies testing the effect of IK14004 and by paired t-test for anti-PD-1 experiments. * P < 0.05, ** P < 0.01, *** P < 0.001, **** P < 0.0001.

Based on peptide-enhanced expression of the IL-12 receptor subunits in CD4+ T cells within TCR-activated splenocyte cultures derived from the LLC model (Fig. 2), we sought to compare these findings with the effect of IK14004 on IL-12 receptor expression in human T cells. Exposure to peptide did not enhance either the proliferative capacity of CD4+ T cells within TCR-stimulated PBMC cultures after 72 h (Fig. 4a) or the proportion of IL-12Rβ1-expressing CD4+ T cells (Fig. 4b) whereas a significant increase in proportion of IL-12Rβ2-expressing CD4+ T cells was observed at the highest peptide concentration (1.25 µM) (Fig. 4c). In CD8+ T cells within stimulated PBMC cultures, IK14004 enhanced the proliferating (Ki67) cell population (Fig. 4d) and this effect was reflected in the non-significant increase in percentage of IL-12Rβ1-expressing CD8+ T cells (Fig. 4e). However, the proportion of IL-12β2-expressing CD8+ T cells nearly quadrupled at the highest peptide concentration compared to vehicle control (Fig. 4f) which could not be accounted for by the relatively small increase in the proliferating CD8+ T cell population (Fig. 4d). We then repeated these studies using non-TCR-activated PBMCs which confirmed an altered IL-12Rβ1: IL-12Rβ2 expression ratio induced by the peptide. For example, expression of IL-12Rβ1 in CD4+ T cells increased approximately two-fold above vehicle control levels in the presence of IK14004 at the highest peptide concentration (Fig. 4g) compared with a four-fold increase in expression of IL-12Rβ2 (Fig. 4h). Similarly, expression of IL-12Rβ1 increased approximately two-fold in CD8+ T cells in the presence of 1.25 µM of peptide (Fig. 4i) compared with a six-fold increase in expression of IL-12Rβ2 at the comparable dose (Fig. 4j). IL-12p70, but not IL-12p40, binds to the IL-12Rβ2 receptor chain^18–20^ and IK14004 preferentially induces IL-12p70 production by T cells^51^. Taken together with the present data showing larger fold increases above vehicle control in IL-12Rβ2 compared with IL-12Rβ1 expression for peptide treated cells, we next tested the effect of anti-PD-1 mAb (10 μg/mL) alone on IL-12 and IFN-γ production in PBMC cultures. Exposure of TCR-stimulated PBMCs to anti-PD-1 did not alter IL-12p70 levels in supernatants after 72 h compared with the isotype control antibody (Fig. 4k). However, anti-PD-1 significantly enhanced production of IL-12p40 (Fig. 4l) and IFN-γ (Fig. 4m) compared with the control antibody.

### IK14004 induces expression of Type I IFNs and activates STAT4 in human T cells

**Figure 5:**
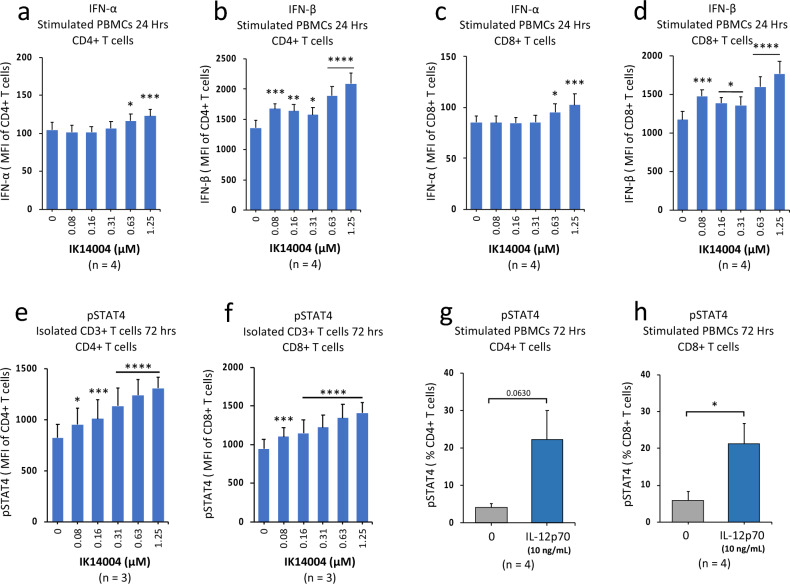
IK14004 induces expression of Type I IFNs and activates STAT4 in human T cells. Buffy coat samples were obtained from human volunteers following ethics approval. PBMC and isolated CD3 + T cell cultures were stimulated with either anti-CD3 or anti-CD3/anti-CD28 antibodies, respectively, and exposed to IK14004 to assess intracellular Type I IFN and pSTAT4 expression after either 24 or 72 h. In separate experiments, anti-CD3-stimulated PBMCs were exposed to recombinant IL-12p70 (10 ng/mL) for 72 h. Each tissue culture experiment was performed using triplicate wells (technical replicates) and repeated at least three or four times (n = experimental replicates) as indicated below each panel. All error bars represent standard error of the mean (SEM). Flow cytometry data are shown as either mean fluorescence intensity (MFI) or percentage of STAT4-expressing cells and dot plot/gating strategies are shown in Supplementary Figs. S9–S11. (**a**) Expression of IFN-α in CD4+ T cells within stimulated PBMC cultures. (**b**) Expression of IFN-β in CD4+ T cells within stimulated PBMC cultures. (**c**) Expression of IFN-α in CD8+ T cells within stimulated PBMC cultures. (**d**) Expression of IFN-β in CD8+ T cells within stimulated PBMC cultures. (**e**) Expression of pSTAT4 in CD4+ T cells within isolated CD3 + T cell cultures. (**f**) Expression of pSTAT4 in CD8+ T cells within isolated CD3 + T cell cultures. (**g**) Expression of pSTAT4 in CD4+ T cells within stimulated PBMC cultures exposed to rIL-12p70. (**h**) Expression of pSTAT4 in CD8+ T cells within stimulated PBMC cultures exposed to rIL-12p70. Data were analysed by two-way ANOVA with Dunnett’s post-test for studies testing the effect of IK14004 and by unpaired t-test in the presence of rIL-12p70. * P < 0.05, ** P < 0.01, *** P < 0.001, **** P < 0.0001.

Productive CD8+ T cell responses require “signal 3” cytokines such as IL-12 or Type I IFNs^54^ which bind to the IL-12 receptor complex with downstream signalling mediated via the IL-12Rβ2 receptor subunit^55^. Taken together with IK14004-mediated induction of IL-12Rβ2 expression (Fig. 4), we questioned whether IK14004 affects Type I IFN expression in T cells. Intracellular expression of Type I IFNs in CD4+ and CD8+ T cells within stimulated PBMCs was assessed by means of flow cytometry following exposure to peptide for 24 h. In CD4+ T cells, a dose-dependent increase at higher peptide concentrations was seen for IFN-α (Fig. 5a) and across all concentrations for IFN-β (Fig. 5b). Similarly, in CD8+ T cells, expression of IFN-α was enhanced in the presence of peptide (Fig. 5c) as was IFN-β (Fig. 5d). Type I IFNs and IL-12 activate STAT4 leading to Th1 differentiation in CD4+ T cells^31^ and Type I IFNs also potentiate human CD8+ T cell-mediated cytotoxicity through STAT4^30^. Hence, we next sought to determine whether IK14004 can enhance STAT4 activation in T cells. Anti-CD3/anti-CD28-stimulated CD3 + T cell cultures were exposed to peptide resulting in marked dose-dependent increases in pSTAT4 expression in CD4+ T cells (Fig. 5e) and in CD8+ T cells (Fig. 5f) after 72 h in culture. Since IK14004 induces IL-12p70 production by T cells^51^, we also examined the effect of recombinant IL-12p70 (rIL-12p70) on STAT4 activation in T cells within TCR-stimulated PBMC cultures. The proportion of pSTAT4-expressing CD4+ T cells increased in the presence of rIL-12p70 after 72 h (Fig. 5g) as did the percentage of pSTAT4-expressing CD8+ T cells (Fig. 5h).

### IK14004 activates natural killer (NK) cells in the absence of IL-2

**Figure 6:**
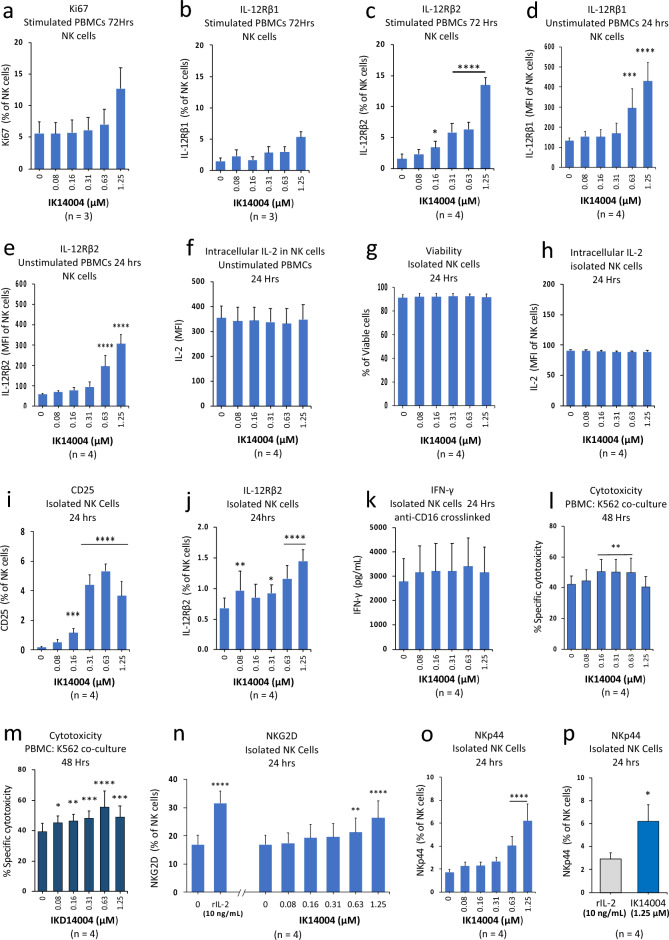
IK14004 activates natural killer (NK) cells in the absence of IL-2. Buffy coat samples were obtained from human volunteers following ethics approval. PBMC cultures exposed to IK14004 were either not stimulated or stimulated with anti-CD3-antibody and cultured for 24 or 72 h, respectively. Cytotoxicity assays in the presence of either IK14004 or the enantiomer, IKD14004, were performed as described in the “Materials and methods”. Isolated CD3−/CD56 + ^dim^ NK cell cultures were cultured for 24 h in the presence of either IK14004 or recombinant IL-2 (10 ng/mL). NK cells were either not stimulated or activated by means of CD16 crosslinking with IgG mAb. Expression of receptors and intracellular IL-2 expression in NK cells was assessed by flow cytometry and IFN- γ production by ELISA. Each tissue culture experiment was performed using triplicate wells (technical replicates) and repeated at least three or four times (n = experimental replicates) as indicated below each panel. All error bars represent standard error of the mean (SEM). Flow cytometry data are displayed as either mean fluorescence intensity (MFI) or percentage of receptor-expressing cells and dot plot/gating strategies are shown in Supplementary Figs. S12–S17. (**a**) Percentage of Ki67-expressing NK cells within stimulated PBMC cultures after 72 h. (**b**) Percentage of IL-12Rβ1-expressing NK cells within stimulated PBMC cultures. (**c**) Percentage of IL-12Rβ2-expressing NK cells within stimulated PBMC cultures. (**d**) Expression levels (MFI) of IL-12Rβ1 in NK cells within unstimulated PBMC cell cultures after 24 h. (**e**) Expression levels (MFI) of IL-12Rβ2 in NK cells within unstimulated PBMC cell cultures. (**f**) Expression levels (MFI) of intracellular IL-2 in NK cells within unstimulated PBMC cultures. (**g**) Viability of isolated NK cell cultures after 24 h. (**h**) Expression levels (MFI) of intracellular IL-2 in isolated NK cell cultures after 24 h. (**i**) Percentage of CD25-expressing NK cells within isolated NK cell cultures. (**j**) Percentage of IL-12Rβ2-expressing NK cells within isolated NK cell cultures. (**k**) IFN-γ production (pg/mL) in supernatant from CD16 crosslinked NK cell cultures. (**l**) K562 cytotoxicity in the presence of IK14004 after 48 h. (**m**) K562 cytotoxicity in the presence of IKD14004 after 48 h. (**n**) Percentage of NKG2D-expressing NK cells within isolated NK cell cultures exposed to either recombinant IL-2 (rIL-2) or IK14004 after 24 h. (**o**) Percentage of NKp44-expressing NK cells within isolated NK cell cultures exposed to IK14004. (**p**) Percentage of NKp44-expressing NK cells within isolated NK cell cultures exposed to either rIL-2 or IK14004. Data were analysed by either one-way or two-way ANOVA with Dunnett’s post-test for studies testing the effect of IK14004 and by paired t-test comparing effects of rIL-2 and IK14004 on NKp44 expression. * P < 0.05, ** P < 0.01, *** P < 0.001, **** P < 0.0001.

In contrast to T cells, IL-2 and not IL-12 is responsible for IL-12 receptor/STAT4 signalling in NK cells^41,42^. Given that IK14004 altered the IL-12Rβ1: IL-12Rβ2 ratio in T cells (Fig. 4), we next examined the response of CD3−/CD56+^dim^ NK cells to peptide. In a preliminary experiment we assessed the proliferative capacity of NK cells within TCR-stimulated PBMC cultures exposed to peptide for 72 h and a non-significant increase in Ki67-expressing cells was observed only at the highest concentration (1.25 μM) (Fig. 6a). Furthermore, the proportion of IL-12Rβ1-expressing NK cells within stimulated PBMC cultures was not significantly altered in the presence of peptide (Fig. 6b) in contrast to a sixfold increase in percentage of IL-12Rβ2-expressing NK cells at the highest peptide concentration (1.25 µM) compared to vehicle control values (Fig. 6c). We then assessed IL-12 receptor expression in NK cells within non-TCR-stimulated PBMC cultures exposed to peptide for 24 h. At a peptide concentration of 1.25 μM, expression of IL-12Rβ1 was enhanced approximately threefold (Fig. 6d) compared with a six-fold increase in IL-12Rβ2 expression (Fig. 6e). To exclude the possibility that NK cell-derived IL-2 might be responsible for these increases in IL-12 receptor expression we then examined the effect of IK14004 on intracellular IL-2 expression in NK cells within unstimulated PBMC cultures and this was not altered in the presence of peptide compared with vehicle control values (Fig. 6f). We next sought to confirm the lack of peptide effect on endogenous IL-2 by exposing isolated NK cells to IK14004 and, in the first instance, assessed the viability of isolated NK cell cultures after exposure to peptide for 24 h. The high percentage of viable NK cells remained unchanged in the presence of peptide at all concentrations (Fig. 6g) as did expression of IL-2 (Fig. 6h).

The high-affinity IL-2 receptor subunit, CD25, is minimally expressed on NK cells and can be induced by IL-12 allowing NK cells to respond to available IL-2^37^. Since IK14004 did not enhance endogenous IL-2 expression within isolated NK cells, we then assessed the effect of IK14004 on expression of CD25 and IL-12Rβ2. Exposure of isolated NK cells to peptide for 24 h induced a dose-dependent increase in the proportion of CD25-expressing NK cells (Fig. 6i) and IL-12β2-expressing cells (Fig. 6j). As part of the cytotoxic response, NK cells produce IFN-γ^56^ and NK cells produce IFN-γ upon activation by crosslinking of the Type III receptor for Fc of IgG (FcγRIII; CD16)^57^. Given the likelihood of not being able to detect secreted IFN-γ by non-activated NK cells, NK cells were activated by cross-linking with anti-IgG mAb in the absence/presence of IK14004 but the peptide did not have an additive effect (Fig. 6k).

We next sought to determine whether IK14004 enhances cytotoxicity of K562 erythroleukemic cells in a K562/PBMC co-culture assay. K562 cells produce significant amounts of aminopeptidases and dipeptidyl-aminopeptidases^58^ and d-amino acids are resistant to most endogenous enzymes^59^. We therefore included the enantiomeric d-amino acid-counterpart of IK14004, i.e., rskaknplyr-4Adods (IKD14004), in this assay. Significant cytotoxicity after 48 h was observed in the presence of the L-isomer, IK14004, at most concentrations (Fig. 6l) and at all concentrations in the presence of IKD14004 (Fig. 6m). IK14004-mediated expression of NKG2D in CD8+ T cells has recently been reported^51^ and the NKG2D-NKG2D ligand interaction in NK cells-mediates killing of K562 cells^60^. The effect of IK14004 on NKG2D expression in unstimulated NK cell cultures was examined next and a dose-dependent increase in the percentage of NKG2D-expressing NK cells was observed after 24 h equivalent to that induced by recombinant IL-2 (10 ng/mL) (Fig. 6n). A characteristic of activated NK cells is expression of the natural cytotoxic receptor, NKp44, that is induced in the presence of IL-2^44^. Hence, we next examined the effect of IK14004 on NKp44 expression in isolated NK cell cultures and IK14004 induced a three-fold increase in the proportion of NKp44-expressing NK cells after 24 h (Fig. 6o). This increase in percentage of NKp44-expressing NK cells in the presence of peptide was significantly greater than that seen in the presence of recombinant IL-2 (10 ng/mL) (Fig. 6p).

## Discussion

In efforts to develop novel agents for cancer immunotherapy that minimise the risk of immune-related adverse events (irAEs) in patients with pre-existing autoimmune diseases^2,3^, we focussed on a lipidic peptide, designated IK14004, that has previously been shown to promote balanced effector-regulatory immune responses^51^. Herein, we demonstrate that IK14004 inhibits LLC progression in a murine model and re-invigorates murine-derived exhausted CD4+ T cells. Moreover, the translational effects in murine splenocytes isolated from the tumour model suggest an indirect immunomodulating role for IK14004 in tumour suppression given lack of effect on the LLC cell line in vitro at concentrations far higher than achievable in vivo after 24 h. Re-invigoration of exhausted T cells is likely to be relevant because murine lung cancer induces generalised T cell exhaustion in both CD4+ and CD8+ T cells^61^.

However, the mechanisms involved in IK14004-mediated inhibition of LLC growth are likely to be multifactorial. Notably, IL-12Rβ1 expression, but not IL-12Rβ2, was enhanced in CD4+ T cells within freshly harvested splenocytes from peptide-treated mice and IFN-γ production was also induced from cultured splenocytes activated with anti-CD3 antibody. Taken together with a positive feed-back loop between IFN-γ and IL-12p40^23,62^ and exclusive binding of IL-12p40 to the IL-12Rβ1 receptor chain^18^, this suggests that IK14004 may alter IL-12 signalling pathways in the LLC model to favour induction of IFN-γ. A limitation of our tumour study is that production of IL-12p40/IL-12p70 by cultured splenocytes was not assessed although IL-12 is thought to be only weakly active in inducing IFN-γ in murine splenocytes^63^. However, signalling through the TCR can induce IL-12Rβ2 expression^64^ which is CD28-dependent^65^. This may explain the increased IL-12Rβ2 expression seen in TCR-activated CD4+ T cells after overnight culture of splenocytes derived from peptide-treated mice on a background of markedly enhanced CD28 expression in CD4+ T cells within freshly harvested splenocytes from mice exposed to peptide (Fig. 2). It is also possible that tumour inhibition is related to peptide-induced expression of IL-12Rβ2 in NK cells given that the IL-12Rβ2 chain is essential for NK cell lysis activity by murine splenocytes^39^. Notably, IL-2 is responsible for upregulating IL-12Rβ2 expression in NK cells^39,41^ through signalling via the IL-2 receptor^66^. Moreover, CD25 expression substantially increases affinity for IL-2 that drives production of lytic molecules^37^. Therefore, we cannot exclude the possibility that enhanced IL-12Rβ2 and CD25 expression in NK cells within freshly harvested splenocytes isolated from peptide-treated mice were not secondary to peptide-induced IL-2 production in vivo.

IL-2 production by TCR-activated human T cells is enhanced in the presence of IK14004^51^ and our finding that IL-2 levels were significantly enhanced in TCR-stimulated splenocyte cultures established from peptide-treated mice compared with control mice suggests that prior exposure to peptide may modulate TCR-responsiveness to TCR stimulation as shown by studies with human PBMCs (Fig. 3). This putative effect may be relatively short-lived in murine splenocytes derived from MBP-tracker mice because enhanced IL-2 production in the presence of IK14004 was observed after 24 but not after 48 h following TCR stimulation with MBP antigen. In contrast, MBP antigen alone at middle and high doses induced further IL-2 production in vehicle control cells after 48 h compared with IL-2 levels after 24 h. We acknowledge that it has not been established whether greater enhancement of IL-2 production might occur when peptide is combined with a suboptimal dose of antigen. This possibility will be investigated in future studies using MBP antigen at a range of doses between 1 and 10 µM and including assay time points earlier than 24 h. Another limitation of our study is that the effects of peptide on expression of TCRα/β subunits in unstimulated human PBMCs were not examined beyond 24 h. This highlights the importance of future studies to assess the kinetics of IL-2 production by splenocytes and tumour-infiltrating lymphocytes across a dose range and after various time points following in vivo peptide administration.

We also acknowledge that our results from murine derived LLC models need to be considered with caution because there are numerous immunological differences between mouse and man of which the complete absence of NKp44 as well as NKG2D ligand differences are just two examples^67^. Nevertheless, data obtained from murine splenocytes and human PBMCs raise the possibility that IK14004 may act in different ways on T cells versus NK cells and we hypothesise that IK14004 acts directly on the TCR to promote IL-2 production. In human T cells, peptide-induced IL-2 production upon TCR stimulation appears to be restricted to CD4+ T cells which may be related to peptide-enhanced expression of TCR α/β subunits in the CD4+ but not CD8+ T cell fraction within unstimulated PBMC populations. In addition, we have not excluded the possibility that the kinetics of TCR stimulation in the presence of peptide may be different between CD4+ and CD8+ T cells despite previous findings that exposure of isolated CD3+ T cells to peptide combined with TCR stimulation for 72 h induces similar levels of Ki67/CD25 expression in both cell subsets^51^. Given that exposure of isolated T cell cultures to IK14004 for 48 h followed by TCR stimulation for a further 48 h induced IL-2 production in CD4+ but not CD8+ T cells (Fig. 3h, i), it will be important in future studies to compare simultaneous versus sequential exposure of T cell subsets to peptide and TCR activation for the same buffy coat samples across a range of timepoints.

In the absence of peptide, we observed that resting, unstimulated T cells have higher expression of IL-12Rβ1 compared with IL-12Rβ2 (Fig. 4). This is consistent with the notion that IL-12Rβ2 appears to be the limiting component of the functional IL-12 receptor^41^. Both IFN-γ and Type I IFNs enhance IL-12Rβ2 expression in CD8+ T cells^39^ and we have previously shown that IK14004 inhibits IFN-γ expression in T cells^51^. Taken together, we propose that IK14004-induced Type I IFN production by TCR-activated PBMCs may be responsible for the larger proportionate increases in IL-12Rβ2 expression above basal levels (vehicle control) at the highest peptide concentration compared with changes in peptide-induced expression levels of IL-12Rβ1. Interestingly, the checkpoint inhibitor anti-PD-1 antibody induces production of IL-12p40, but not IL-12p70, together with enhanced IFN-γ expression. In contrast, IK14004 only enhances IL-12p70 but not IL-12p40 expression in isolated T cell cultures and inhibits IFN-γ production^51^. For patients with pre-existing autoimmune diseases, the use of ICIs is associated with significant risk of irAEs^68^. Hence, many clinical trials exclude cancer patients with pre-existing autoimmune diseases^69^. Notably, 14–25% of patients with lung cancer suffer from autoimmune diseases^70,71^ and exactly how IFN-γ regulates the balance between protection and development of autoimmune responses remains to be determined^68^.

Both IL-12 receptor subunits are required for binding to IL-12p70^12^ and we suggest that IK14004 favours enhanced binding of IL-12p70 over IL-12p40 to the heterodimeric IL-12 receptor (IL-12Rβ1/IL-2Rβ2) secondary to the relatively greater fold-increase in IL-12Rβ2 expression above basal levels compared with IL-12Rβ1. Furthermore, IL-12p70 signalling via IL-12Rβ2-mediated STAT4 activation induces IL-12Rβ2 transcription^19^. This is in line with our findings of inducible STAT4 activation in T cells by recombinant IL-12p70 and the dose-dependent increases in phosphorylation of STAT4 in CD4+ /CD8+ T cells in the presence of peptide. Moreover, activation of STAT4 by IFN-α, but not IFN-γ, induces tyrosine phosphorylation and DNA binding of STAT4^72^ and IK14004 induces expression of Type I IFNs. Importantly, STAT4 has been associated with favourable prognoses for several cancer types^73^ while also not appearing to be the culprit gene in promoting autoimmune responses in mouse models^74^.

While activated NK cells produce IFN-γ^56^, we also confirmed that IK14004 does not induce IFN-γ production upon activation of NK cells by means of CD16-crosslinking and does not induce IL-2 expression in isolated NK cells or NK cells within unstimulated PBMC cultures. Nevertheless, IK14004 enhanced expression of CD25 and IL-12Rβ2 (Fig. 6) and IL-12 receptor expression in NK cells is known to be IL-2-dependent^41,42^. Furthermore, neither endogenous IL-12 nor Type I/II IFNs are considered responsible for IL-12Rβ2 expression in NK cells^39^. This begs the question of how IK14004 activates NK cells? Expression of the natural cytotoxic receptor, NKp44, in NK cells is dependent on IL-2^44^ and IK14004 enhanced the proportion of NKp44-expressing NK cells in isolated NK cell cultures significantly more than recombinant IL-2. This suggests that isolated NK cells cultures were unlikely to be contaminated with IL-2-expressing CD4+ T cells and that IK14004 appears to act as an IL-2-like “influence factor”. Whether peptide plus recombinant IL-2 (rIL-2) can exert an additive, or even synergistic, effect on activation of NK cells warrants further investigation aided by transcriptomic and epigenetic profiles of treated cells. It is also possible that lymphocyte-specific protein tyrosine kinase (Lck) could be involved in this process as seen with upstream signalling upon TCR activation^75,76^ that leads to IL-2 production^77^. For example, NK cells express phospho-Lck (pLck)^78^. Notably, Lck is phosphorylated by IL-2 in human NK cells^79^ and IK14004 has been shown to phosphorylate Lck in non-cell-based assays^51^. Taken together with involvement of pLck in NKG2D-mediated cytotoxicity^78^ and IK14004-mediated induction of NKG2D expression in NK cells, this highlights the relevance of assessing Lck activity in NK and CD8+ T cells exposed to peptide in future studies.

The NKG2D receptor recognises ligands usually absent on normal cells but upregulated by stress signals^48^ associated with various NKG2D-ligand-expressing tumour cells^11,80,81^. Upregulation of NKG2D in CD8+ T cells occurs via signalling through the IL-12Rβ2/STAT4 receptor complex^45^ and CD8+ T cell effector functions do not develop in the absence of a “third signal” such as IL-12 or Type I IFN^82^. IK14004 inhibits IFN-γ production in CD8+ T cells^51^ in contrast to stimulating Type I IFN expression and Type I IFNs regulate STAT4 activation in CD8+ T cells^30^ which may explain why IK14004 activates STAT4 in CD8+ T cells. To add to this complexity, IL-12 signalling in CD8+ T cells is regulated by both Type I/II IFNs in a STAT4-dependent manner^39,40^ and STAT4 can act not only as a transcriptional promoter but also as a repressor within the same cell^31^. Taken together, gene profiling studies in the presence of IK14004 may provide more mechanistic information on this complex behaviour.

We do not know whether IK14004-mediated cytotoxic effects in human K562 cells are due to CD8+ T cells and/or NK cells. Notably, an increased proportion of granzyme B-expressing CD8+ T cells in the presence of peptide indicates CD8+ T cell activation and IK14004 also enhances expression of NKG2D in CD8+ T cells^51^. NKG2D-NKG2D-ligand interactions in NK cell-mediated killing activity of K562 cells are abolished by NKG2D-blocking antibody^60^ and another limitation of our study is that effects of IK14004 in K562 co-culture assays were not assessed in the presence of NKG2D-blocking antibody. While IFN-γ is unlikely to be involved in IK14004-mediated K562 cytotoxicity, peptide-mediated expression of IL-12 receptors in NK cells could be expected to facilitate IL-12 signalling. IL-12-mediated cytolysis of tumour cells via increased expression of NKG2D in NK cells is associated with production of cytotoxic effector molecules such as TRAIL and perforin^83^ and for NK cells to be fully functional, activation by either Type I IFNs or pro-inflammatory cytokines such as IL-12 is required^84^. K562 cells also express ligands for NKp44^85^. Hence, a possible role for IK14004-enhanced NKp44 expression in NK cells contributing to K562 cytotoxicity cannot be dismissed. However, this remains a complex matter to resolve because tumours can exploit NKp44 to escape NK cell recognition via expression of proliferating cell nuclear antigen to inhibit NK cell effector function^86^ and target cell lysis by NKp44 occurs only in combination with other activating receptors^87^**.**

In summary, IK14004 modulates upstream signalling at the TCR and activates NK cells in an IL-2-like manner leading to expression of activating receptors which may play a role in cytotoxicity against cancer cells. Peptide-mediated IL-12 receptor changes in T cells and NK cells suggest skewed expression in favour of IL-12Rβ2 expression over IL-12Rβ1. Notably, consumption of IL-12 by IL-12Rβ2-expressing Tregs not only reduces the availability of IL-12 to Th1 effector cells but also enhances the Treg immunosuppressive effect which may have a therapeutic advantage in suppressing Th1-mediated autoimmunity^88^. While the relevance of increases in the IL-12Rβ2 : IL-12Rβ1 receptor chain ratio to prevention of autoimmune responses remains to be established, IL-12Rβ2 has been shown to offer protection against both spontaneous autoimmunity and malignancy in murine models^25,28^. Taken together with contrasting effects between the peptide^51^ and anti-PD-1 antibody on IL-12p40, IL-12p70 and IFN-γ production, this serves to highlight the important role of IL-12Rβ2 in the regulation of IL-12 responsiveness^24^. IFN-γ-mediated resistance to anti-PD-1 therapy is well-recognised^89^ and together with exacerbation of autoimmune pathologies these remain critical unmet needs associated with ICI therapy. The novel peptide offers an opportunity to gain further insight into the complexity of ICI immunotherapy so that autoimmune responses may be minimised without promoting tumour evasion from the immune system.

## Materials and methods

*Human Ethics* All methods were carried out in accordance with relevant guidelines and regulations. Buffy coat samples from healthy human donors were obtained from Research Donors Limited via Cambridge BioScience. Ethics approval was granted by the Black Country Research Ethics Committee under REC reference 19/WM/0260. Informed consent for buffy coat samples was obtained from all subjects and/or their legal guardians in accordance with the Helsinki Declaration.

*Animal Ethics* Murine splenocyte assays, Lewis lung cancer (LLC) growth studies and pharmacokinetic analyses.

*Accordance* All methods were carried out in accordance with relevant guidelines and regulations.

*Arrive guidelines* All methods are reported in accordance with ARRIVE guidelines (https://arriveguidelines.org).

For murine splenocyte assays (exhausted and non-exhausted CD4+ T cells) and Lewis lung cancer (LCC) studies, animal procedures were conducted within the animal facility at the University of Edinburgh in procedure rooms designated for use by Concept Life Sciences (CLS). The University of Edinburgh and CLS are committed to the highest standards of animal welfare and are subject to legislation under the Animals (Scientific Procedures) Act 1986. All studies were conducted in accordance with the Act, with UK Home Office Guidance on the implementation of the Act and with all applicable Codes of Practice for the care and housing of laboratory animals.

Peptide biodistribution studies in mice were approved by the University of Queensland Animal Ethics Committee and all studies were in accordance with guidelines of the Animal Ethics Committee of The University of Queensland (UQ; Approvals AIBN/530/15/ARC/NHMRC and 2020/AE000044) and the Australian Code for the Care and Use of Animals for Scientific Purposes.

### Peptide synthesis

The lipidic peptides IK14004 [sequence: RSKAKNPLYR-(2)Adod-(2)Adod-(2)Adod-(2)Adod-amide] and IKD14004 [sequence: rskaknplyr-(2)Adod-(2)Adod-(2)Adod-(2)Adod-amide] were manufactured by Auspep (Melbourne, Australia) using solid phase peptide synthesis with Fmoc protected amino acid building blocks. The core [(2)Adod]_4_-resin was synthesised by sequentially coupling four (2)Adod [(S)-2-aminododecanoic acid] residues (Watanabe Chemical Industries LTD, Japan) onto a Rink AM resin. The IK14004 and the IKD14004 sequences were then assembled in turn by adding sequentially either the L, or D, isomeric form of the protected amino acid respectively onto the core [(2)Adod]_4_-resin. Once synthesis was completed the resin peptides were globally deprotected and cleaved from the resin liberating the crude, C-terminally amidated lipidic peptides. These were purified, and salt exchanged to acetate by RP-HPLC (C18) to a purity of > 95%. The product structures were confirmed by mass spectroscopy and amino acid analyses.

### In vitro cell culture studies

Preparations of peripheral blood mononuclear cells (PBMCs) and isolated T cells from buffy coat samples were performed using SepMate tubes, EasySep selection and enrichment kits, Lymphoprep, RoboSep Buffer, and EasySep magnets (STEMCELL Company). PBMCs were resuspended in RPMI-10 (RPMI-1640; ThermoFisher) supplemented with 10% heat inactivated Foetal Bovine Serum (LabTech), 100 U/mL penicillin, 100 µg/mL streptomycin (ThermoFisher), 2 mM l-glutamine (ThermoFisher), and 50 µM β-mercaptoethanol (ThermoFisher) at 1 × 10^6^ cells/mL and plated at a density of 1 × 10^5^ per well (100 µL) in 96-well, flat-bottom culture plates. PBMCs were tested either unstimulated or stimulated with 1 µg/mL of soluble anti-CD3 (BioLegend) and isolated T cell cultures stimulated with anti-CD3/anti-CD28 coated Dynabeads (ThermoFisher). The lipidic peptide IK14004 and its D-enantiomer (IKD14004), were solubilised as a 1 mM stock solution in sterile milliQ water (Lonza) and added to wells at a final volume of 50 µL per well. Peptide concentrations ranged from 0.08 to 1.25 µM. Vehicle controls in peptide-based experiments comprised 0.13% sterile milliQ water in culture medium.

CD3+ T cells were isolated from PBMCs by negative selection using immune-magnetic separation (Stem cell kits), resuspended in complete medium as used for PBMCs at 0.5 × 10^6^/mL and plated at a density of 5 × 10^4^ per well (100 µL) in 96-well, flat bottom culture plates. CD4+ and CD8+ T cell populations were isolated by immunomagnetic separation and resuspended in RPMI-10 at 0.5 × 10^6^/mL with a plating density of 0.5 × 10^5^ per well. Peptide IK14004 was added to wells at a final volume of 50 µL per well, together with anti-CD3 anti-CD28 coated Dynabeads (ThermoFisher) at a 4:1 cell:bead ratio (1.25 × 10^4^/well, 50 µL volume) and cells cultured for 72 h at 37 °C and 5% CO_2_.

In parallel PBMC and CD3+ T cells culture assays, cells were cultured for 48 h at 37 °C and 5% CO_2_, after which 2 stimulation approaches were adopted; (1) IK14004 was washed out with fresh medium after a 48 h pre-activation period followed by TCR activation for a further 48 h or (2) IK14004 remained in culture prior to and during anti-CD3 (1 µg/mL) stimulation (BioLegend, lot no. B225107) for PBMCs and anti-CD3/anti-CD28 stimulated CD4+ /CD8+ T cell cultures that commenced at the 48 h timepoint. Hence, the total culture period for both approaches was 96 h and IL-2 was assessed by ELISA (R&D Biotechne, lot no. P100155).

CD3−/CD56+^dim^ NK cell populations were isolated from PBMCs using immuno-magnetic separation (Stem cell, Lot no. 18A86421). CD56^+^ NK cells were resuspended in RPMI-10 (RPMI-1640 supplemented with 10% heat inactivated FBS, 100 U/mL penicillin, 100 µg/mL streptomycin, 2 mM l-glutamine, and 50 µM β-mercaptoethanol) at 0.25 × 10^6^/mL and plated at a density of 2.5 × 10^4^ per well (100 µL) in 96-well, flat bottom culture plates. Purified NK cells were cultured together with either IK14004 or recombinant IL-2 (InVitrogen, Lot #223086-011) for 24 at 37 °C, 5% CO_2_.

### In vitro assays

#### Flow cytometry

Staining was performed to determine cell viability (Flexible Viability Dye eFluorTM 780; ThermoFisher) and expression of extracellular/intracellular markers assessed using fluorescently-labelled antibodies against human proteins within different cell populations. T cells and NK cells (CD3^neg^CD56^+^) were assessed for the following markers: CD4 (FITC Mab OKT4; ThermoFisher, BV421 Mab OKT4; BioLegend), CD8 (BV711/clone SK1, BioLegend), CD25 (PE/Cy7, PerCP/Cy5.5; BioLegend), IL-12Rβ1/IL-12Rβ2 (PE, IL-12Rβ1/ PE, APC, IL-12Rβ2; BioTechne (R&D Systems), NKp44 (PerCP/Cy5.5; BioLegend) and NKG2D (PE; BioLegend) and in intracellular staining studies using PBMC and isolated NK cell preparations, Brefeldin A (3 μg/mL) (Life Technologies, Cat #00-4506-51, Lot #2229153) was added to the cell cultures 4 h prior to flow cytometry. Intracellular expression of either Ki67 (AF-488; BioLegend) or IFN-α and IFN-β (PE; BD Bioscience and FITC; BioTechne, respectively) was determined for CD4^+^/CD8^+^ T cell fractions within PBMC cultures and viability of NK cells assessed (Flexible Viability Dye eFluorTM 780; ThermoFisher) and intracellular expression of IL-2 in NK cells (PE; BioLegend) within cultures of either unstimulated PBMCs or isolated NK cells. To assess TCRα/β expression, PBMCs were stimulated with anti-CD3 (1 µg/mL, 50 µL volume) (BioLegend, Lot no. B238685) or left unstimulated and cultured in the presence of IK14004 at a single concentration (50 µL volume). After 24 h cells were collected and CD4+ and CD8+ T cells assessed for TCRα/β expression (PE; BioLegend). Phospho-STAT4 in was assessed in TCR-activated CD3 + T cell and PBMC cultures. In the former, cells were exposed to IK14004 for 72 h and then fixated using the BD Phosflow™ Fix buffer I (BD Bioscience) and permeabilised to allow for intracellular staining using BD Phosflow™ Perm Buffer III (BD Bioscience). Cells were then stained with fluorochrome conjugated anti-pSTAT4 antibody (eBioscience, Lot# 12-9044) and intracellular expression determined within CD8+ T cells by flow cytometry. Similarly, anti-CD3-stimulated PBMC cultures were exposed only to recombinant IL-12p70 (Peprotech, Lot #0617596) and pSTAT4 assessed within CD4+ /CD8+ T cell fractions. Anti-PD-1 and IgG1 antibodies were obtained from BioLegend and recombinant IL-2/IL-12p70 from Peprotech. Flow cytometry data were exported as FCS files from Attune™ NxT software and analysed using FlowJo™ software.

#### ELISA assays

Culture supernatants were obtained from unstimulated and anti-CD3-stimulated PBMCs, anti-CD3/anti-CD28-stimulated CD4+ /CD8+ T cells and CD16 crosslinked NK cells. Production of IL-2 and IFN-γ (ThermoFisher kits) was assessed for PBMC/T cells and NK cells, respectively. In separate experiments, NK cells were incubated with anti-CD16 mAb (ThermoFisher, Lot #4341947) (10 µg/mL) for 30 min. Cells were cultured with goat anti-mouse IgG1 F(ab’)2 (ThermoFisher, Lot #BVH3063301) (10 µg/mL) +/− IK14004 for 24 h. At the end of culture period, supernatants were collected and assessed for IFN-γ by ELISA (Invitrogen, Lot #221271-010). ELISA plates were read at 450 nm using an Infinite F50 (Tecan) absorbance reader and Magellan™ reader control and data analysis software.

#### Cytotoxicity assay

Human PBMCs (2.5 × 10^5^ cells per well) were cultured in 96-well round-bottom tissue culture plates for 48 h at 37 °C, 5% CO_2_ in the presence of IK14004 and IKD14004 peptides. After peptide exposure, PBMCs were co-cultured with Calcein AM stained K562 cells (5 × 10^4^/well) at a 5:1 ratio for 4 h at 37 °C, 5% CO_2_. For spontaneous release control Calcein AM-K562 cells alone were treated with PBS (20 µL). For maximal release control Calcein AM-K562 cells alone were treated with lysis buffer (20 µL). After 4 h culture, 150 µL supernatant were transferred to black wall plates and intensity of Calcein green fluorescence release detected using GloMax® Discover to determine percentage of specific cytotoxicity.

#### Cancer cell proliferation assay

Lewis lung cancer (LLC) cells (sourced from the American Type Culture Collection) were seeded into 96-well plates (1000 cells/well) in complete cell culture medium and allowed to attach for 24 h (37 °C, 5% CO2 in air). Next, an equal volume of either cell culture medium only, or 2 × concentration of drug dissolved in cell culture medium, was added to each of 5 replicate wells (technical replicates) to expose cells to concentrations of IK14004 in the dose range from 0 to 5 µM. Cells were cultured for 72 h in the presence of either IK14004 or Doxorubicin (2.5 µM; positive control) and then the cell culture medium was removed and the attached cells fixed in ice-cold trichloroacetic acid. Fixed cells were stained with Sulforhodamine B (SRB) and then washed with 1% acetic acid to remove unbound dye. The retained dye was solubilised in 10 mM Tris base solution and the absorbance at 550 nm was measured with the baseline (media only without cells) subtracted. The data were normalised between the maximum proliferation (100%, cells with no drug) and the starting cell density (0%, cells before addition of drug). Each experiment was performed on two independent occasions (biological replicates).

#### Murine splenocyte-derived exhausted CD4+ T cell assay

Concept Life Sciences have established a proprietary method for assessing murine CD4+ T cell exhaustion in vitro using Tg4-Ly5.1 MBP-Tracker Mice obtained from the University of Edinburgh. Spleens were removed from transgenic B10PLxC57BL/6 mice and processed to generate a single cell suspension of splenocytes. Myelin basic protein (MBP)-Tracker splenocytes were resuspended at 3 × 10^6^/mL and stimulated 72 h at approximately 37 °C, 5% CO_2_, with altered peptide ligand (APL)-MBP (to generate exhausted cells). Following stimulation, T cells were purified by Ficoll density gradient, and subsequently re-plated at 2 × 10^6^/mL in 20 U/mL IL-2 for four days. At the end of this rest period, cells were resuspended (4 × 10^5^/mL, final 2 × 10^4^ per well) and restimulated using irradiated APCs (from B10PLxC57BL/6 mice, 4 × 10^6^/mL, final concentration of 2 × 10^5^ cells per well) plus a single dose of APL-MBP peptide together with IK14004 peptide for 72 h. At the end of splenocyte culture periods and prior to commencing cytokine/receptor assays, cells were routinely checked at the highest concentration (1.25 µM) and no visual signs of cell death were evident.

Supernatants were sampled from culture wells for assessment of IL-2 and IFN-γ by ELISA (eBioScience, Lot # 4280695 and Lot #. 4308729, respectively) and flow cytometry used for assessment of CD25 and Ki67 (eBioScience, Lot # 4276862 and Lot # 4296884, respectively). Anti-PD-1 and IgG2a antibodies were obtained from BioXCell.

#### Murine splenocyte-derived non-exhausted CD4+ T cell assay

MBP-Tracker mouse studies were conducted using Tg4 Ly5.1 (MBP-Tracker Mouse), B10PlxC57BL/6 mice for spleen harvesting for the naïve cell assay. A single spleen per experiment was removed from MBP-Tracker mice and each processed to generate single cell suspensions of splenocytes. The splenocytes were cultured at 3 × 10^5^/well and stimulated with myelin basic protein (MBP) peptide antigen at three doses, 0.1, 1.0 and 10 μg/mL, together with IK14004 for a period of 48 h. Supernatants were collected at 24 and 48 h and assessed for IL-2 production by ELISA (eBioscience lot no. 4280695) according to manufacturer’s instructions.

### Biodistribution studies

#### Labelling

1,4,7-Triazacyclononane-1,4,7-triacetic acid (NOTA) conjugated peptides were acquired from Auspep, reconstituted in deionised water at 10 mg/mL and used without further purification. Peptides were incubated with ^64^Cu at 1000-fold excess of peptide in 0.1 M pH 5.5 ammonium acetate buffer for 45 min at 37 °C. 1 µL Samples of each solution were taken and mixed 1:1 with 50 mM EDTA. EDTA incubated sample or neat solution was spotted on TLC paper (Agilent iTLC-SG Glass microfiber chromatography paper impregnated with silica gel) and run with 50:50 H_2_O:ethanol. Radioactivity in the TLC was then measured utilizing an Eckert and Zeigler Mini-Scan and Flow-Count (B-MS-1000F) radio-TLC detector. Control experiments were conducted to monitor the elution profile of free ^64^Cu (R_f_ 0) and ^64^Cu bound to EDTA (R_f_ 1) for quality control. All samples showed 100% radiolabelling purity. Labelled peptides were then added to stocks of unlabelled peptide in deionised water to achieve a mass dose of 200 μg peptide and approximately 3 MBq [^64^Cu]NOTA-peptide) per 50 μL injection per C57BL/6 mouse.

#### Dosing

Mice were anaesthetised using 2% isofluorane in O_2_ for all injection and imaging procedures throughout. Either male Balb/C or female C57BL/6 mice (approximately 8 weeks of age) were injected (29G needle, 50 µL aqueous solution) with labelled peptide (200 μg) either via the tail vein or intraperitoneally, respectively. Activity present in the injection syringe was measured prior to dosing and the calculated injected dose determined from the residual after injection and used for calculation of percentage injected dose/g (%ID/g). Peptide uptake in the lungs, spleen and heart of Balb/C mice was assessed in vivo during the first 4 h following injection and images were acquired using a Siemens Inveon PET-CT scanner. In the ex vivo study using C57BL/6 mice, animals were sacrificed by cervical dislocation after 24 h. Blood and tissues were collected and the tissues cleaned of excess blood and weighed for ex vivo analysis. A PerkinElmer 2480 Automatic Gamma Counter was used to measure radioactivity in tissues. The gamma counter was calibrated using known samples of ^64^Cu and measured activity presented as %ID/g based on injected activities.

### Lewis lung cancer (LCC) tumour studies

#### Allografts

LLC cells sourced from ATCC. The Lewis lung cancer cell line was cultured to approximately 70% confluency before cells were collected and resuspended at 5 × 10^6^ /mL in sterile HBSS. B16F10/6 mice were injected subcutaneously with 5 × 10^5^ cells (100 µL) into the right flank. Mice were randomly assigned to treatment groups 5 days after tumour cell implantation such that the average tumour size was approximately equal between the two groups. Test substance IK14004 or vehicle were administered twice weekly (Monday and Thursday) via intraperitoneal (i.p.) injections, from day 5 post tumour cell implantation, until the tumours reached an average of 10 mm in diameter in the vehicle treated group. Tumours were measured three times each week using digital callipers and once the mean tumour size for the vehicle group reached 10 mm in diameter, mice were sacrificed by cervical dislocation and tumour and spleens collected. Spleens were processed into single cell suspensions and the red blood cells lysed. Fresh splenocyte suspensions were assessed by trypan blue exclusion. From fresh splenocyte suspensions, 1 × 10^6^ viable cells were stained using a viability dye (Flexible Viability Dye eFluorTM 780; ThermoFisher), CD4 (BV605), NK1.1 (BV510), CD25 (AF488), IL-12Rβ1 (PE) and IL-12Rβ2 (APC) for flow cytometry.

#### Lung metastases

LLC cells sourced from ATCC. Following a week of acclimatisation, the C57BL/6 mice were randomly assigned to a treatment group whilst ensuring that mice in each cage were in receipt of the same treatment to eliminate the potential for any cross contamination. On Day 0 mice received 0.5 × 10^6^ LLC cells by intravenous tail vein injection. Treatments were given twice weekly I.P. (Days 1, 4, 8 and 11 post cell transfer). Mice were weighed recorded at the start of the study and monitored daily. Fifteen days post cell transfer, mice were euthanised by schedule 1 method (rising CO_2_ concentration). Lungs were removed and placed in Bouin’s solution to fix and stain the lung tissue to distinguish tumours. Surface tumour metastasis was not done due to the diffuse nature of a number of visible metastases and it was determined that a better assessment was possible with histological analysis of haematoxylin and eosin stained sections as per the Concept Life Sciences Histoplex protocol.

Spleens were collected and processed into a single cell suspension. Fresh splenocytes were assessed by means of flow cytometry with CD28 (BV421) and 2 × 10^5^ cells per well were then cultured overnight with or without TCR stimulation (anti-CD3 alone or in combination with anti-CD28 both at 2 µg/mL, plate bound overnight in PBS at 4 °C). After overnight culture cell free supernatants were collected and assessed for IFN-γ and IL-2 by ELISA (eBioscience Kits) and set up in technical triplicate. Cells before and after stimulation were stained for flow cytometry (viability, CD4, CD28 (BV711), IL-12Rβ1 and IL-12Rβ2.

### Statistical analyses

For flow cytometry and ELISA experiments, data from IK14004 and vehicle groups were analysed using parametric statistical procedures. Data within groups to be compared were assumed to be normally distributed and to satisfy the homogeneity of variance criterion. Statistical analyses for flow cytometry/ELISA assays were performed using GraphPad Prism (version 8.4.2) on a Windows Operating System. Data from groups in LLC studies were analysed by unpaired two-tailed t test. Data from in vitro studies were analysed by means of either one-way or two-way ANOVA with Dunnett’s post-test for peptide dose/vehicle comparisons and Sidak’s post-test for multiple comparisons between groups. Single compound effects, e.g., anti-PD-1 mAb versus isotype control antibody, or single dose recombinant cytokine/IK14004 peptide/vehicle control comparisons were analysed by paired t-test.

### Supplementary Information

Supplementary Figures.

## Data Availability

Dot plot/gating strategies for flow cytometry studies are available in the Supplementary Information provided. All remaining raw datasets used and/or analysed during the current study are available from the corresponding author on reasonable request.
