# Supplementary material for: An immunomodulating peptide with potential to suppress tumour growth and autoimmunity
Source: Sci Rep. 2023 Nov 13;13:19741. doi: 10.1038/s41598-023-47229-y (PMC10643673; doi:10.1038/s41598-023-47229-y)
Supplement: Supplementary file 1 — Supplementary Figures. [file 41598_2023_47229_MOESM1_ESM.pdf]

An immunomodulating peptide with potential to suppress tumour growth and autoimmunity

Michael Agrez[1,4]*, Christopher Chandler[2], Kristofer J. Thurecht[3,4], Nicholas L. Fletcher[3,4], Feifei Liu[3,4], Gayathri Subramaniam[3,4], Christopher B. Howard[3,4], Benjamin Blyth[5], Stephen Parker[1], Darryl Turner[6], Justyna Rzepecka[6], Gavin Knox[6], Anastasia Nika[6], Andrew M. Hall[6], Hayley Gooding[6] & Laura Gallagher[6]

[1]InterK Peptide Therapeutics Limited, New South Wales, Australia. [2]Auspep Pty Limited, Melbourne, Australia. [3]Centre for Advanced Imaging, University of Queensland, Australia. [4]Australian Institute for Bioengineering and Nanotechnology and the ARC Training Centre for Innovation in Biomedical Imaging Technologies, University of Queensland, Australia. [5]Peter MacCallum Cancer Centre and Sir Peter MacCallum Department of Oncology at the University of Melbourne, Melbourne, Australia. [6]Concept Life Sciences, Edinburgh, Scotland.

Supplementary information
Flow cytometry data

Figure S1: Refers to manuscript **Figure 1d and 1e**
CD25 and Ki67 (Splenocyte-derived exhausted CD4+ T cells)

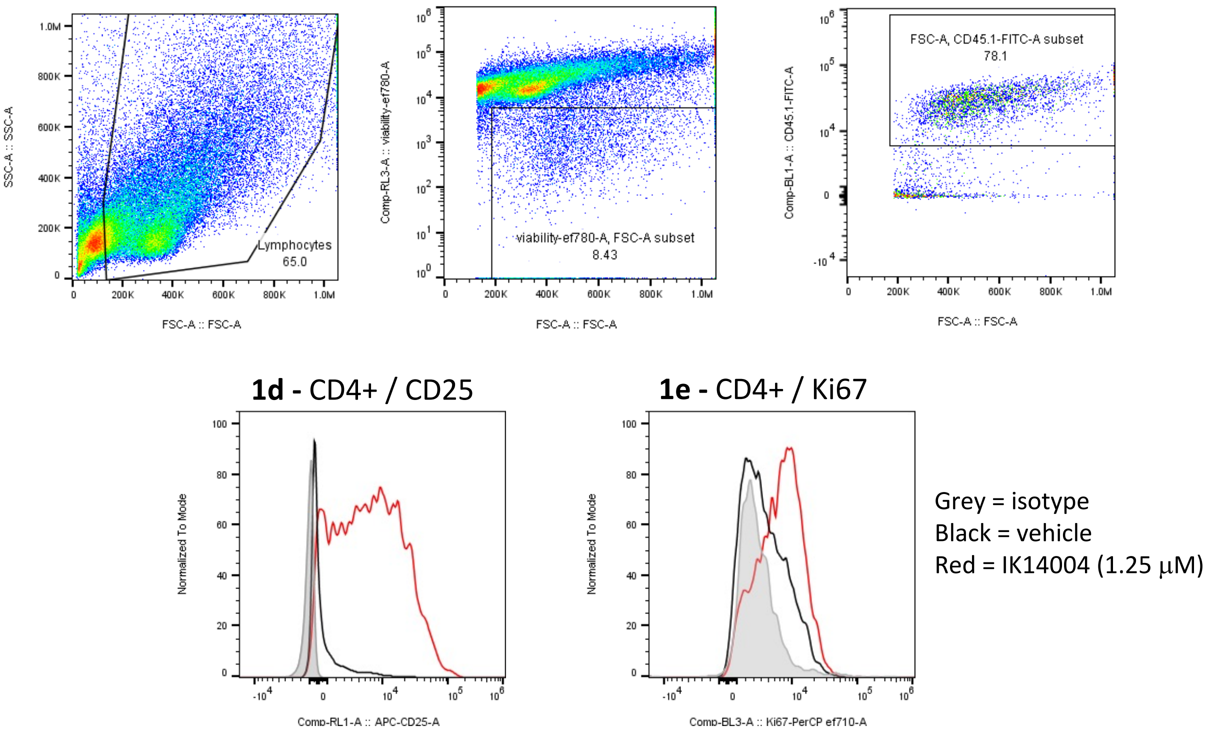

The Figure shows an exemplar gating strategy from Donor 1. Debris were excluded, then viable (live) and CD45.1+ cells (Tg4 exhausted CD4+ T cells) selected for analysis of CD25 (1d) or Ki67 (1e).

Figure S2: Refers to manuscript **Figure 2e – 2k**

CD25, IL-12Rβ1 and IL-12Rβ2 (Splenocytes derived from the LLC xenograft model CD4+ T cells)

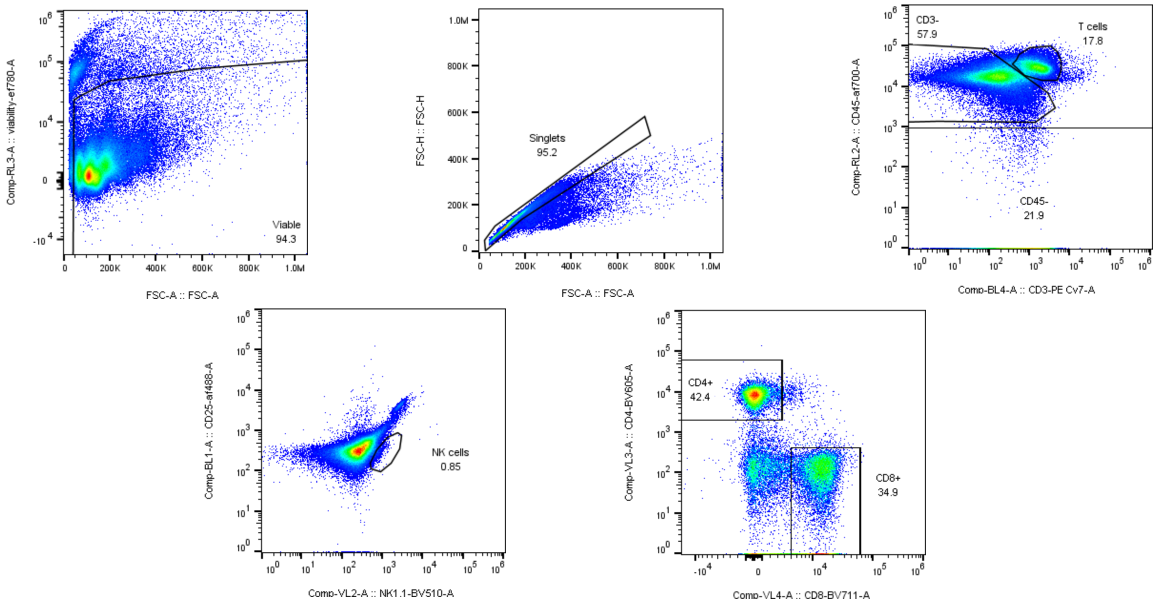

The Figure shows an exemplar gating strategy from mouse 2.0 (IK14004). Viable (live) cells selected, doublets excluded, $CD45^{+}CD3^{-}$ and $CD45^{+}CD3^{+}$ cell populations selected. Within $CD3^{-}$ cells, $NK1.1^{+}$ cells were selected and within $CD3^{+}$ cells, $CD4^{+}$ and $CD8^{+}$ cell populations gated.

Viability

IK14004

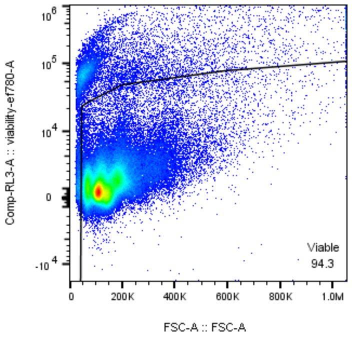

Vehicle

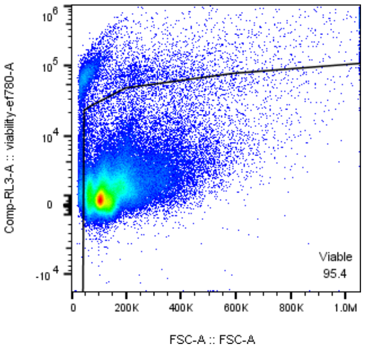

Figure S2: Refers to manuscript **Figure 2e – 2k (continued)**
CD25, IL-12Rβ1 and IL-12Rβ2 (Splenocytes derived from the LLC xenograft model CD4+ T cells)

2f - CD4+ / CD25 | **2g -** CD4+ / IL-12Rβ1 | **2h -** CD4+ / IL-12Rβ2

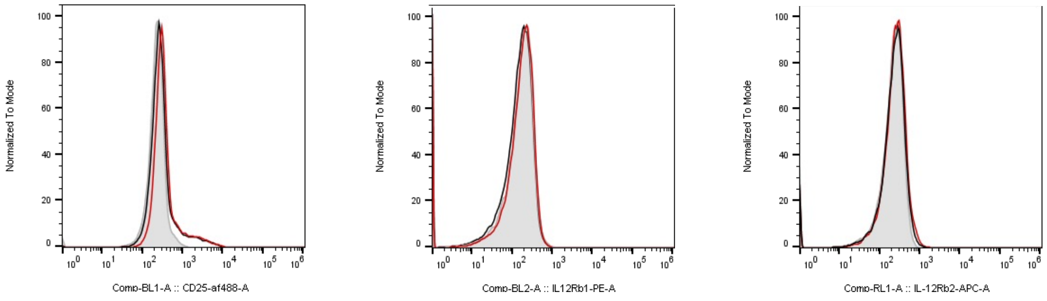

Grey = isotype
Black = vehicle
Red = IK14004

Analysis of CD25, IL-12Rβ1 and IL-12Rβ2 in CD4⁺ T cells.

2f - NK1.1+ / CD25 | **2g -** NK1.1+ / IL-12Rβ1 | **2h -** NK1.1+ / IL-12Rβ2

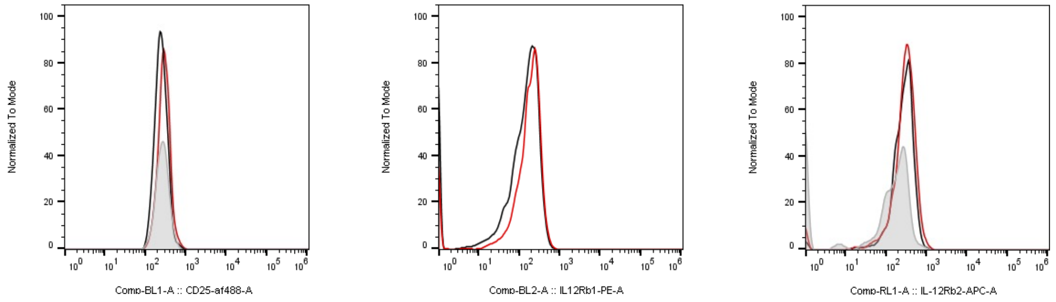

Grey = isotype
Black = vehicle
Red = IK14004

Analysis of CD25, IL-12Rβ1 and IL-12Rβ2 in NK1.1⁺ cells.

Figure S3: Refers to manuscript **Figure 2m**

CD28 (Splenocytes derived from the LLC xenograft model CD4+ T cells)

Non-stimulated splenocytes

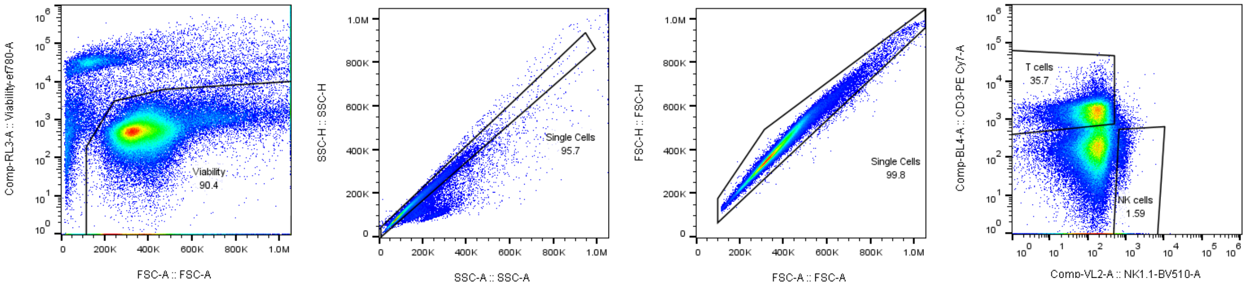

2m - CD4+ / CD28

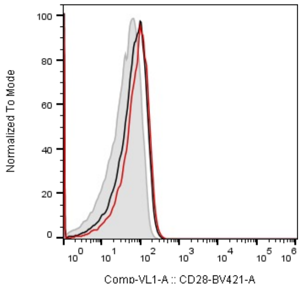

Grey = isotype
Black = vehicle
Red = IK14004

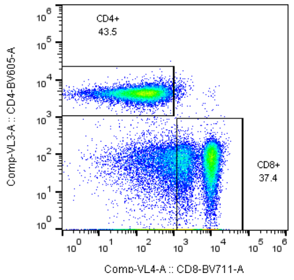

The Figure shows an exemplar gating strategy from mouse 2.0 (IK14004). Viable (live) cells selected, doublets excluded, $CD3^+$ T cell and $CD3^-NK1.1^+$ NK cell populations selected. Within $CD3^+$ cells, $CD4^+$ selected for analysis of CD28.

Figure S4: Refers to manuscript **Figure 2n, 2q and 2r**

Viability, IL-12Rβ1 and IL-12Rβ2 (Cultured splenocytes derived from the LLC xenograft model CD4+ T cells)

Viability: cultured splenocytes

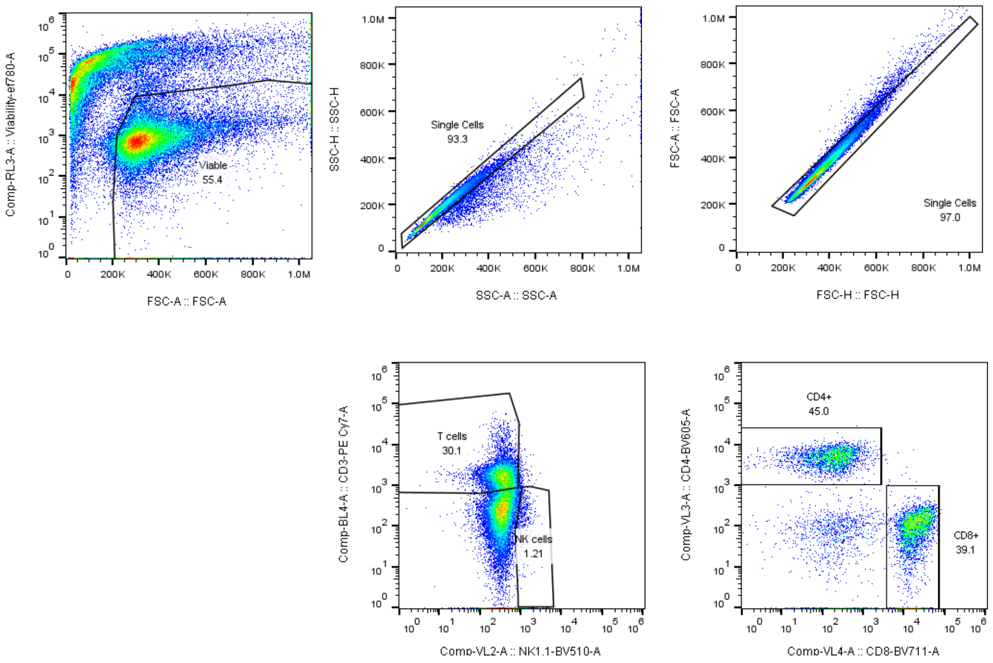

2n - Viability

Unstimulated (IK14004) CD3 (IK14004) CD3/CD28 (IK14004)

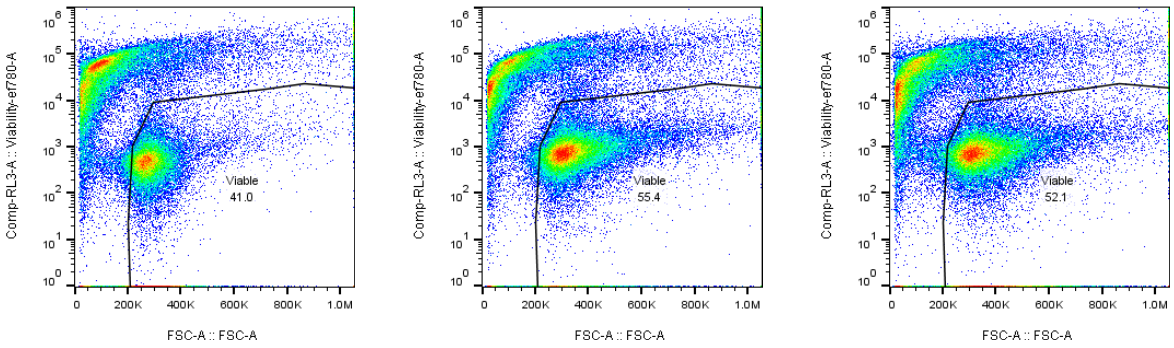

Figure S4: Refers to manuscript **Figure 2n, 2q and 2r (continued)**
IL-12Rβ1 and IL-12Rβ2 (Cultured splenocytes derived from the LLC xenograft model CD4+ T cells)

2q - CD4+ / IL-12Rβ1

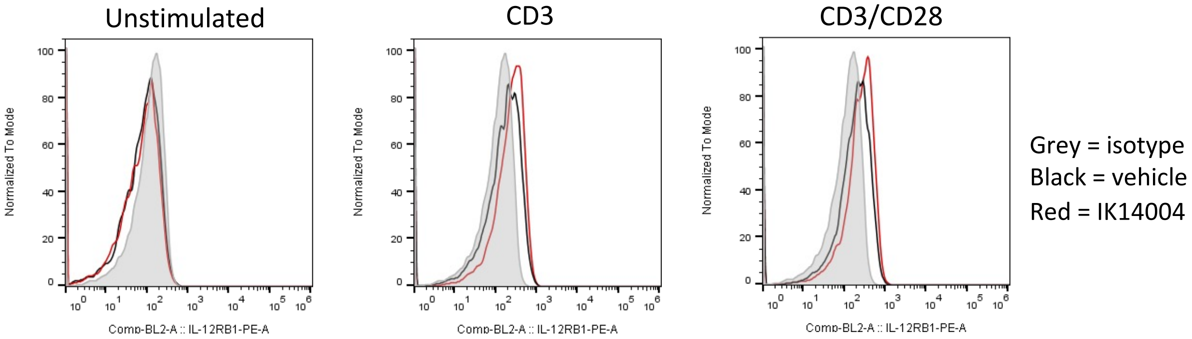

2r - CD4+ / IL-12Rβ2

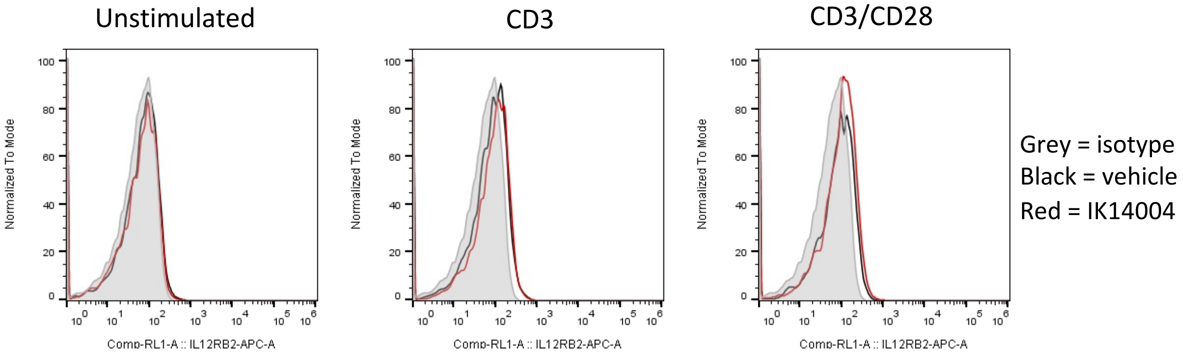

The Figure shows an exemplar gating strategy from anti-CD3 stimulated mouse 2.0 (IK14004). Viable (live) cells selected, doublets excluded, $CD3^{+}$ T cell and $CD3^{-}NK1.1^{+}$ NK cell populations selected. Within $CD3^{+}$ cells, $CD4^{+}$ selected for analysis of IL-12Rβ1 and IL-12Rβ2.

Figure S5: Refers to manuscript **Figure 3a and 3e**
Ki67 and CD25 (Unstimulated human PBMC cultures)

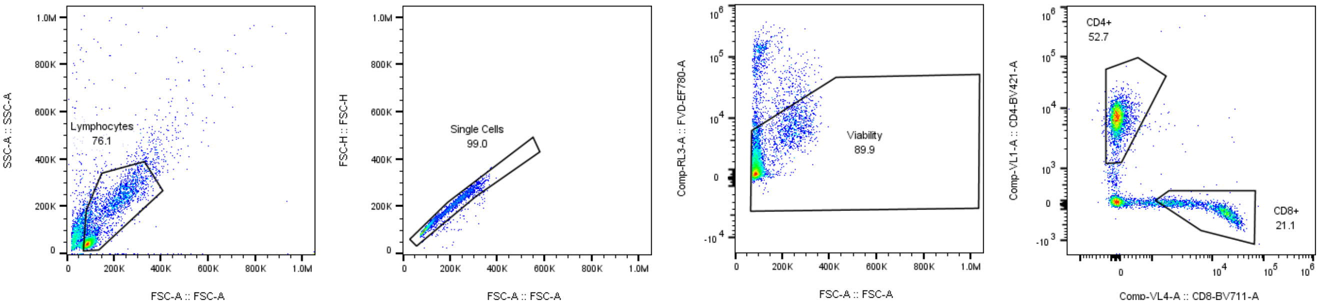

3a - CD4+ / Ki67

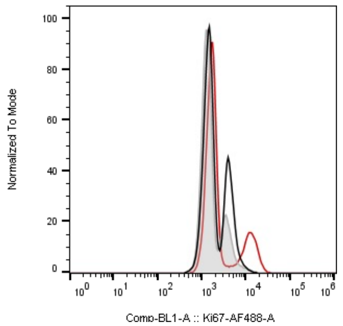

Grey = isotype
Black = vehicle
Red = IK14004 1.25 µM

3e - CD4+ / CD25

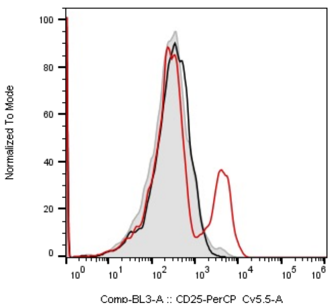

Grey = isotype
Black = vehicle
Red = IK14004 1.25 µM

The Figure shows an exemplar gating strategy from Donor 3 unstimulated PBMCs (IK14004 1.25 µM). Cells gated to remove debris, doublet cell exclusion, and viable (live) cells selected. Within viable cells, $CD4^{+}$ T cell selected for analysis of Ki67 and CD25.

Figure S6: Refers to manuscript **Figure 3j and 3k**

CD4+/CD8+ **-** TCRα/β (Unstimulated/stimulated human PBMC cultures)

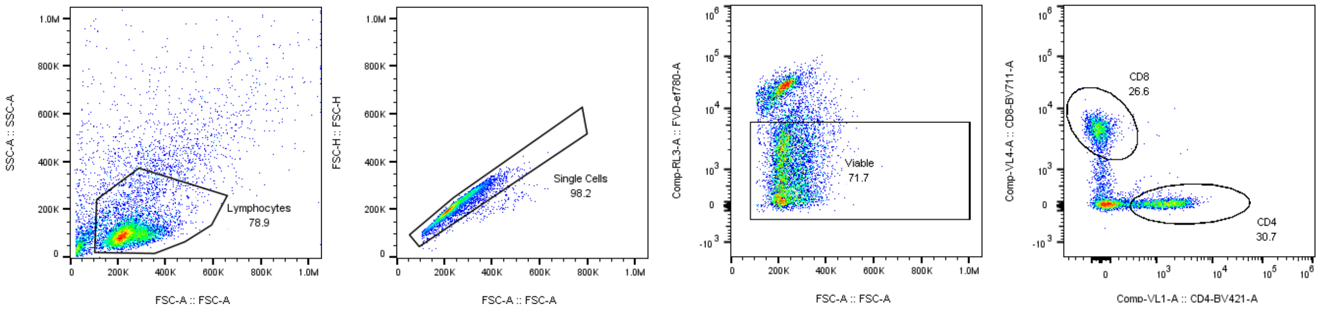

3j - CD4+ / TCRα/β

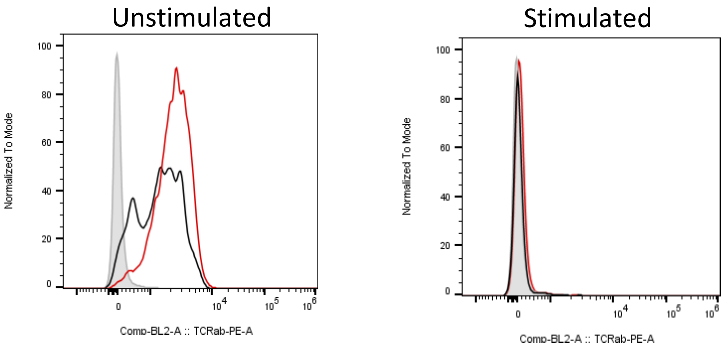

Grey = isotype

Black = vehicle

Red = IK14004 1.25 µM

3k - CD8+ / TCRα/β

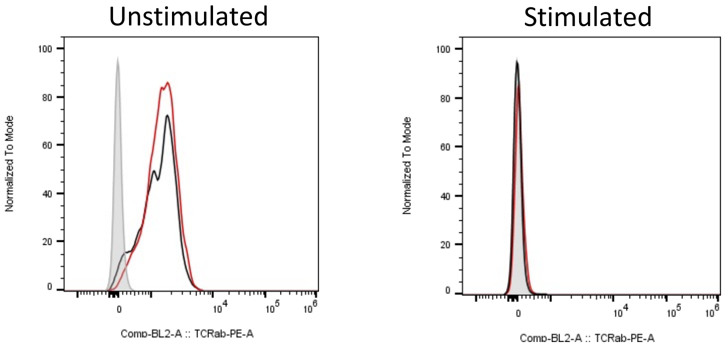

Grey = isotype

Black = vehicle

Red = IK14004 1.25 µM

The Figure shows an exemplar gating strategy from Donor 1 unstimulated PBMCs (IK14004 1.25 µM). Cells gated to remove debris, doublet cell exclusion and viable (live) cells selected. Within viable cells, $CD4^+$ and $CD8^+$ T cells selected for analysis of TCRα/β.

Figure S7: Refers to manuscript **Figure 4a – 4f**

Ki67, IL-12Rβ1 and IL-12Rβ2 (Stimulated human PBMC cultures: CD4+/CD8+ T cells)

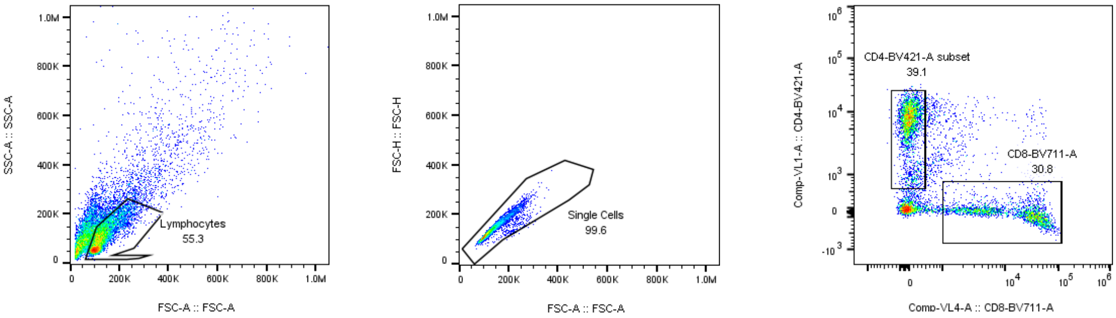

The Figure shows an exemplar gating strategy from Donor 2 stimulated PBMCs (IK14004 1.25 µM). Cells gated to remove debris, doublet cell exclusion and CD4⁺ and CD8⁺ T cells selected for analysis of Ki67, IL-12Rβ1 and IL-12Rβ2.

4a - CD4+ / Ki67

IK14004 1.25 µM   Vehicle

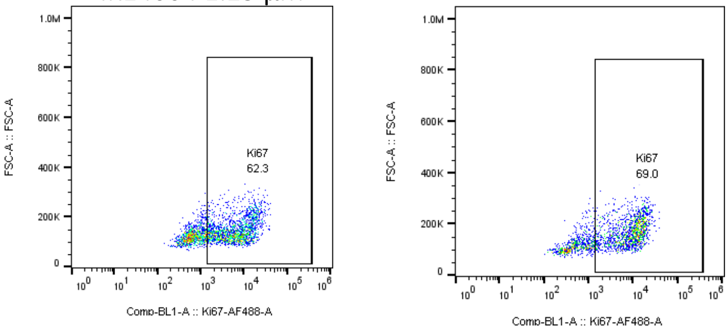

4b - CD4+ / IL-12Rβ1

IK14004 1.25 µM   Vehicle

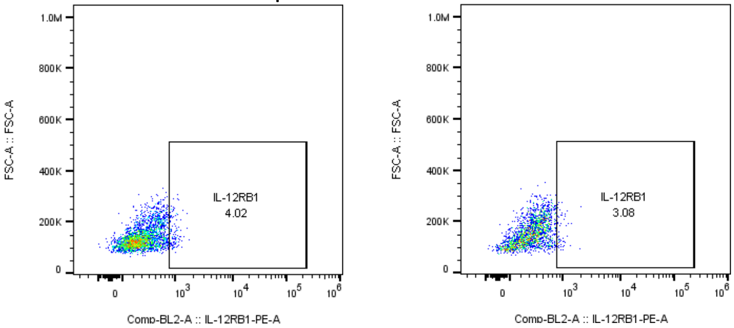

4c - CD4+ / IL-12Rβ2

IK14004 1.25 µM   Vehicle

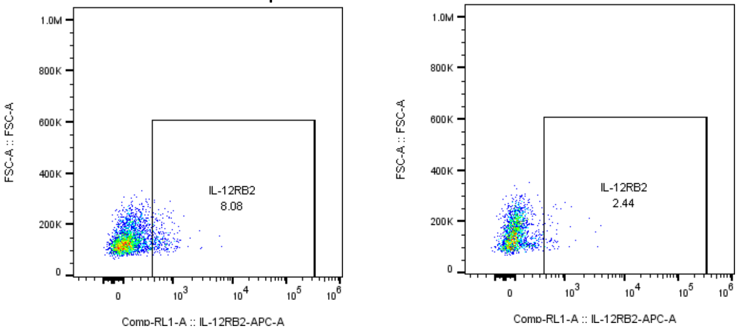

Figure S7: Refers to manuscript **Figure 4a – 4f (continued)**
Ki67, IL-12Rβ1 and IL-12Rβ2 (Human PBMC cultures: CD4+/CD8+ T cells)

4d - CD8+ / Ki67

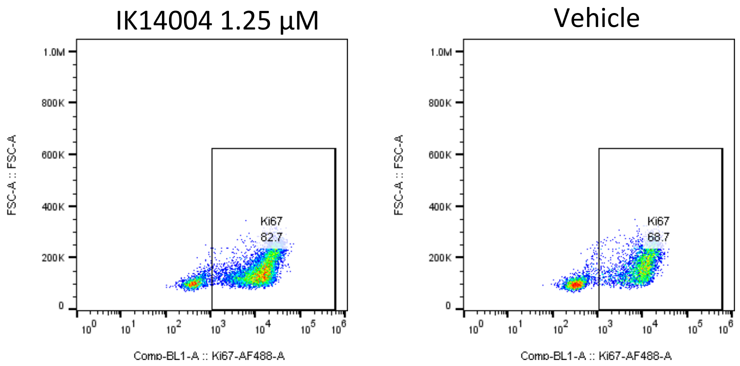

4e - CD8+ / IL-12Rβ1

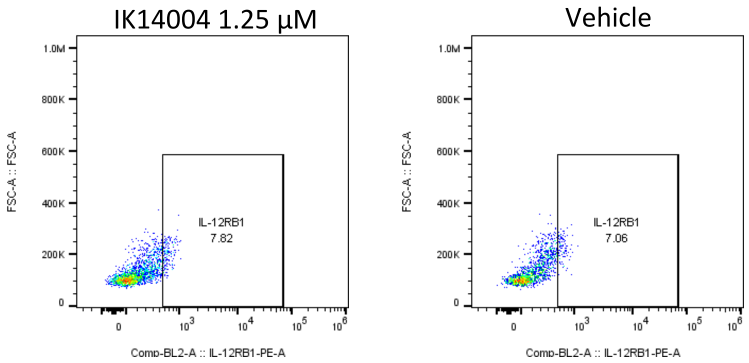

4f - CD8+ / IL-12Rβ2

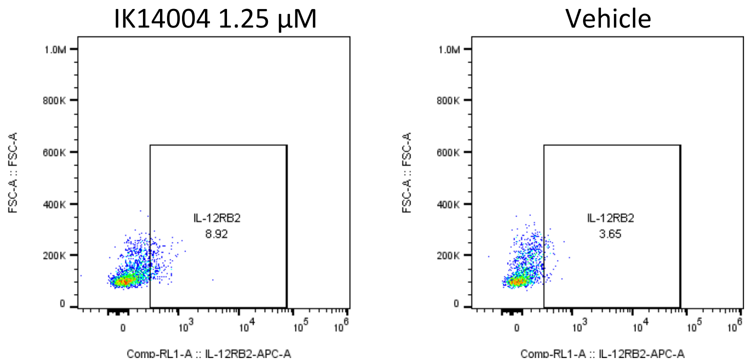

Cells gated to remove debris, doublet cell exclusion and $CD4^+$ and $CD8^+$ T cells selected for analysis of Ki67, IL-12Rβ1 and IL-12Rβ2 (Donor 2 and 3).

Figure S8: Refers to manuscript **Figure 4g – 4j**
IL-12Rβ1 and IL-12Rβ2 (Unstimulated human PBMC cultures: CD4+/CD8+ T cells)

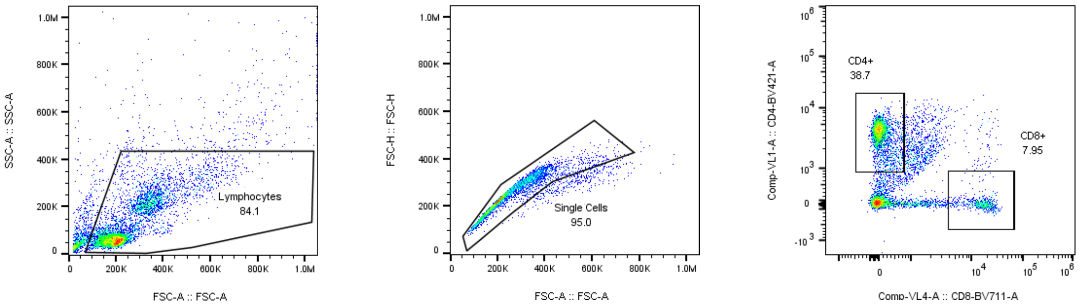

4g - CD4+ / IL-12Rβ1

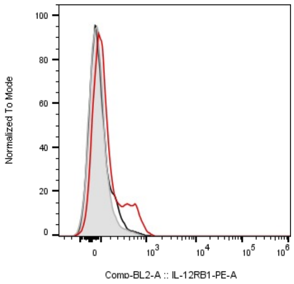

4h - CD4+ / IL-12Rβ2

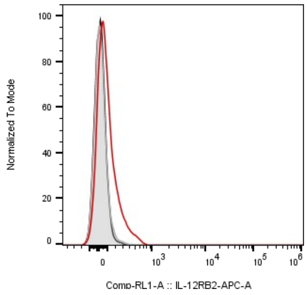

Grey = isotype
Black = vehicle
Red = IK14004 1.25 µM

4i - CD8+ / IL-12Rβ1

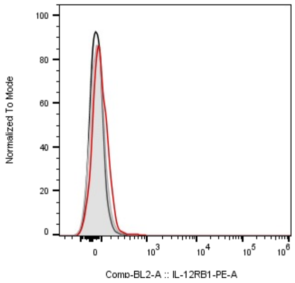

4j - CD8+ / IL-12Rβ2

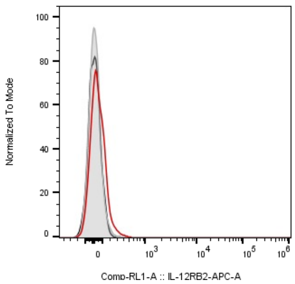

Grey = isotype
Black = vehicle
Red = IK14004 1.25 µM

The Figure shows an exemplar gating strategy from Donor 1 unstimulated PBMCs (IK14004 1.25 µM). Cells gated to remove debris, doublet cell exclusion and $CD4^+$ and $CD8^+$ T cells selected for analysis of IL-12Rβ1 and IL-12Rβ2.

Figure S9: Refers to manuscript **Figure 5a - 5d**

IFN-α and IFN-β (Stimulated human PBMC cultures: CD4+/CD8+ T cells)

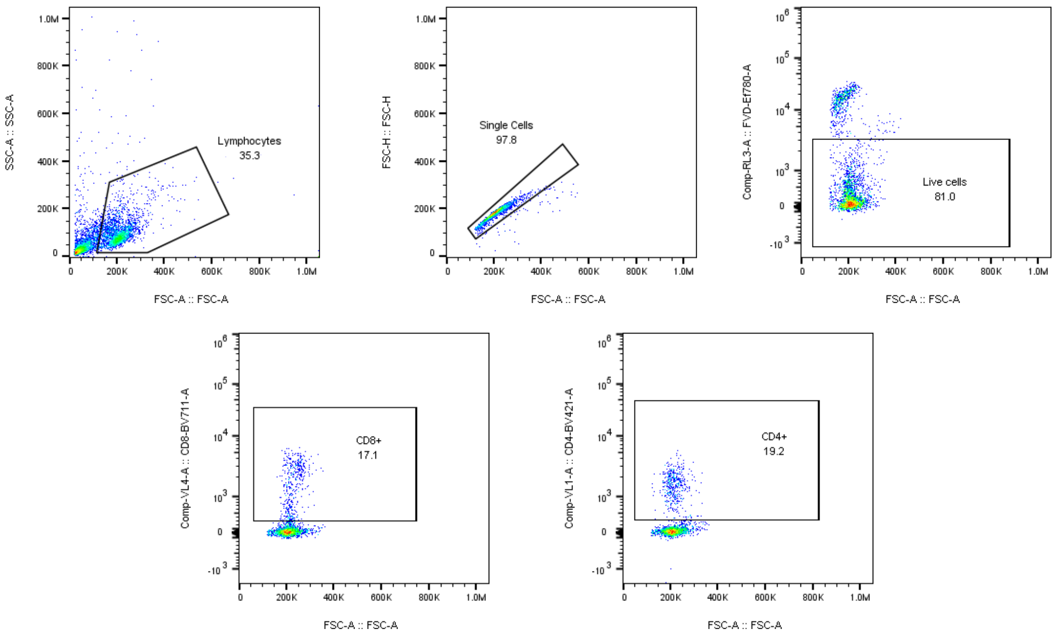

5a - CD4+ / IFN-α

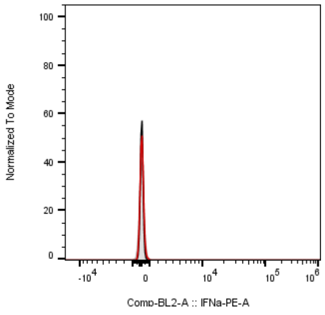

5b - CD4+ / IFN-β

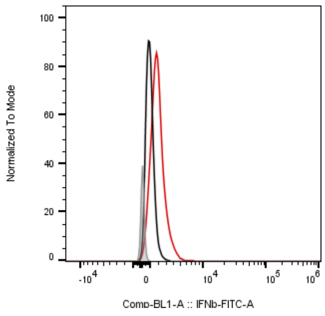

Grey = isotype
Black = vehicle
Red = IK14004 1.25 µM

5c - CD8+ / IFN-α

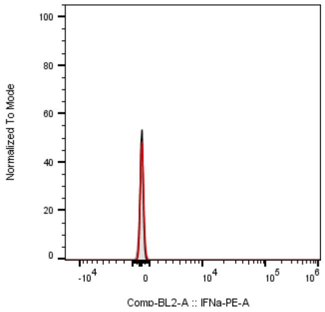

5d - CD8+ / IFN-β

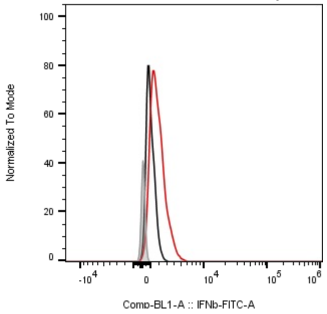

Grey = isotype
Black = vehicle
Red = IK14004 1.25 µM

The Figure shows an exemplar gating strategy from Donor 2 stimulated PBMCs (IK14004 1.25 µM). Cells gated to remove debris, doublet cell exclusion, viable cells gated and $CD4^+$ and $CD8^+$ T cells selected for analysis of IFN-α and IFN-β.

Figure S10: Refers to manuscript **Figure 5e and 5f**
pSTAT4 (Isolated, stimulated human CD3+ T cells)

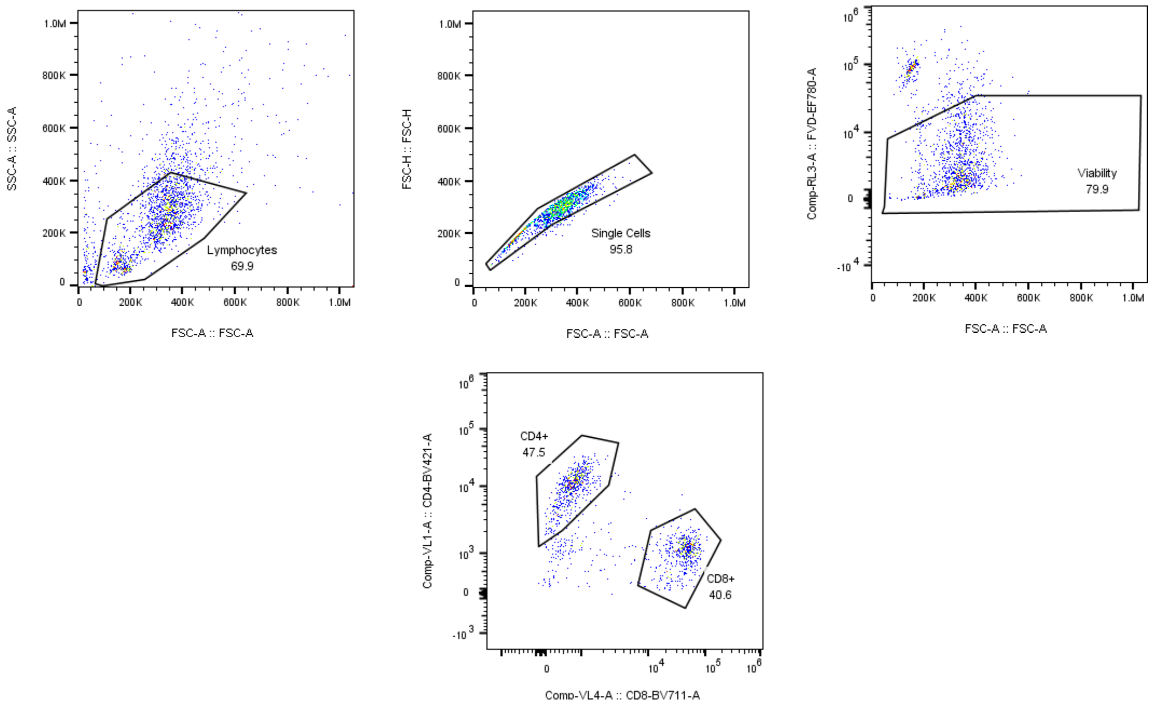

5e - CD4+ / pSTAT4

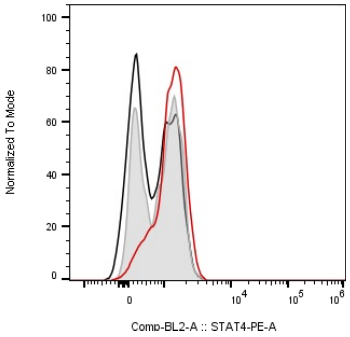

5f - CD8+ / pSTAT4

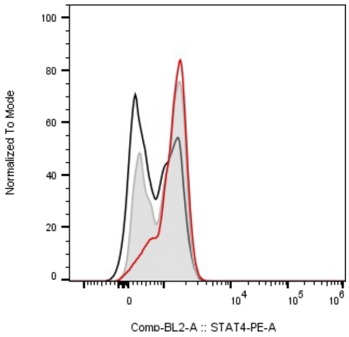

Grey = isotype
Black = vehicle
Red = IK14004 1.25 µM

The Figure shows an exemplar gating strategy from Donor 1 stimulated CD3^{+} T cells (IK14004 1.25 µM). Cells gated to remove debris, doublet cell exclusion, viable cells gated and CD4^{+} and CD8^{+} T cells selected for analysis of pSTAT4.

Figure S11: Refers to manuscript **Figure 5g and 5h**
pSTAT4 (Stimulated human PBMC cultures: CD4+/CD8+ T cells)

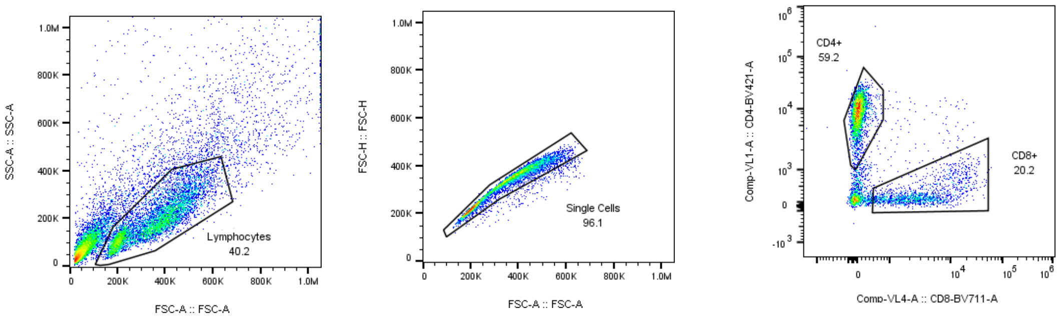

5g - CD4+ / pSTAT4

Grey = isotype
Red = IL-12p70

Grey = isotype
Red = vehicle

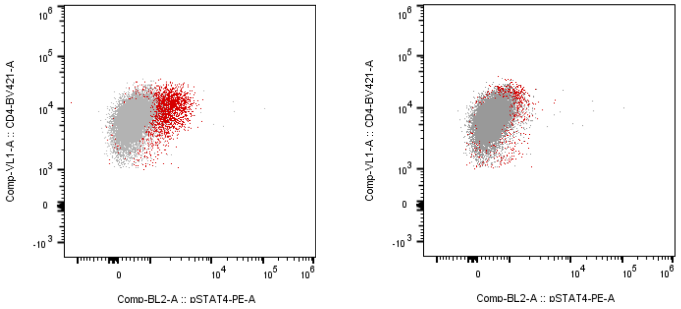

5h - CD8+ / pSTAT4

Grey = isotype
Red = IL-12p70

Grey = isotype
Red = vehicle

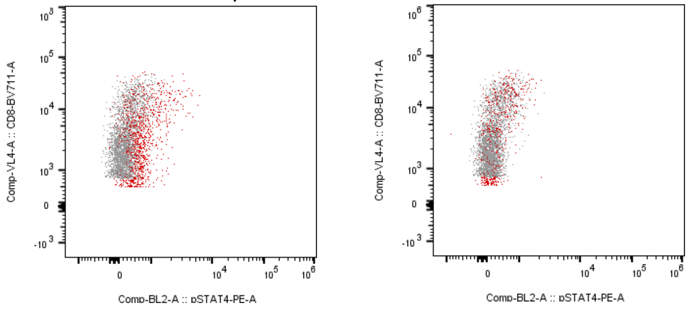

The Figure shows an exemplar gating strategy from Donor 1 stimulated PBMC (IL-12p70). Cells gated to remove debris, doublet cell exclusion, and CD4⁺ and CD8⁺ T cells selected for analysis of pSTAT4.

Figure S12: Refers to manuscript **Figure 6a - 6c**

Ki67, IL-12Rβ1 and IL-12Rβ2 (Stimulated human PBMC cultures: CD3-CD56+^{dim} NK cells)

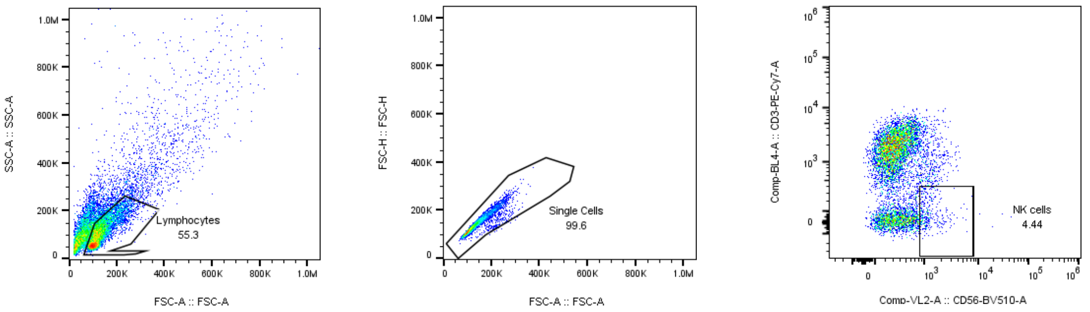

6a - CD3-CD56+^{dim} / Ki67

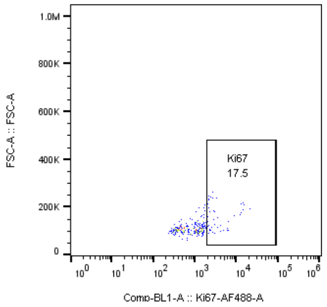

6b - CD3-CD56+^{dim} / IL-12Rβ1

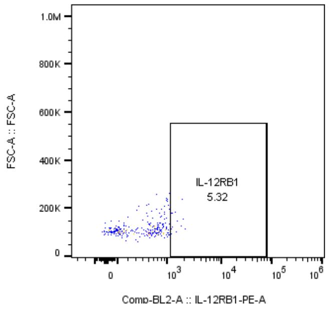

6c - CD3-CD56+^{dim} / IL-12Rβ2

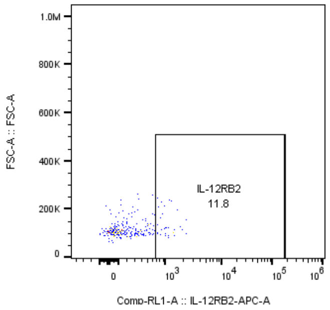

The Figure shows an exemplar gating strategy from Donor 3 stimulated PBMC (IK14004 1.25 µM). Cells gated to remove debris, doublet cell exclusion, and CD3-CD56+^{dim} NK cells selected for analysis of Ki67, IL-12Rβ1 and IL-12Rβ2 (IK14004 1.25 µM).

Figure S13: Refers to manuscript **Figure 6d and 6e**

IL-12Rβ1 and IL-12Rβ2 (Unstimulated human PBMC cultures: CD3-CD56+^{dim} NK cells)

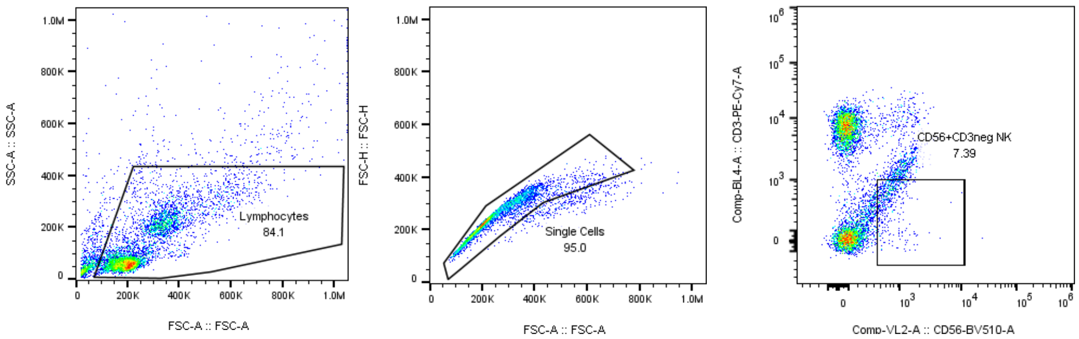

6d - CD3-CD56+^{dim} / IL-12Rβ1

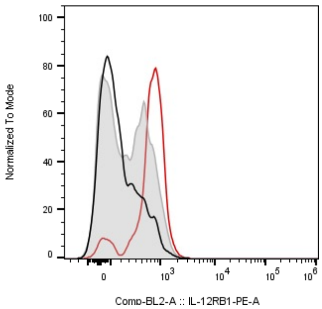

Grey = isotype
Black = vehicle
Red = IK14004 1.25 µM

6e - CD3-CD56+^{dim} / IL-12Rβ2

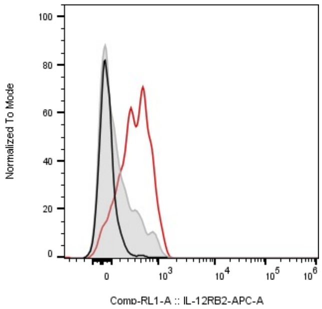

Grey = isotype
Black = vehicle
Red = IK14004 1.25 µM

The Figure shows an exemplar gating strategy from Donor 1 unstimulated PBMC (IK14004 1.25 µM). Cells gated to remove debris, doublet cell exclusion, and CD3-CD56+^{dim} NK cells selected for analysis of IL-12Rβ1 and IL-12Rβ2.

Figure S14: Refers to manuscript **Figure 6f**

Intracellular IL-2 (Unstimulated human PBMC cultures)

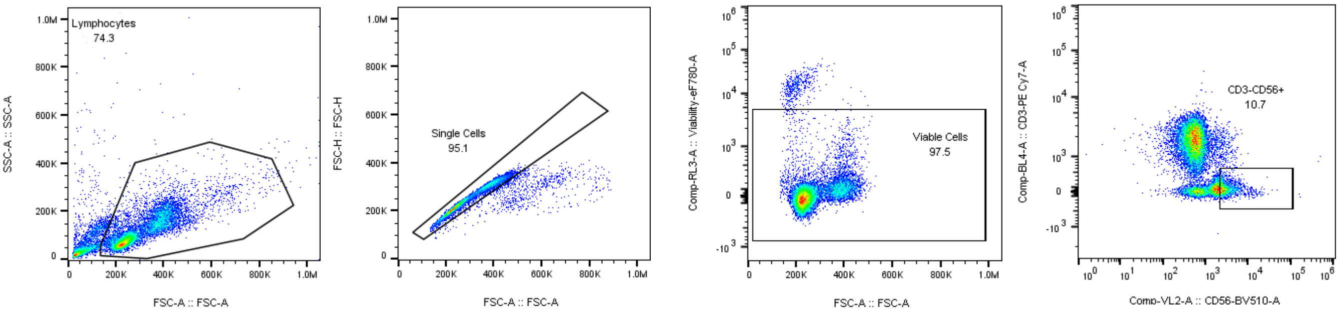

6f - CD3-CD56+^{dim} / IL-2

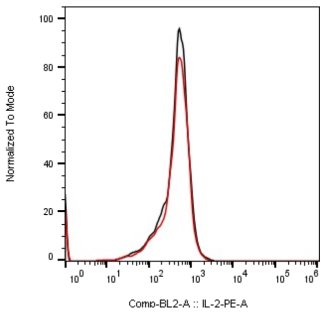

Black = vehicle

Red = IK14004 1.25 µM

The Figure shows an exemplar gating strategy from Donor 3 unstimulated PBMC (IK14004 1.25 µM). Cells gated to remove debris, doublet cell exclusion, viable cells gated and CD3-CD56+^{dim} NK cells selected for analysis of intracellular IL-2.

Figure S15: Refers to manuscript **Figure 6g and 6h**
Viability and Intracellular IL-2 (Isolated CD3-CD56+^{dim} NK cells)

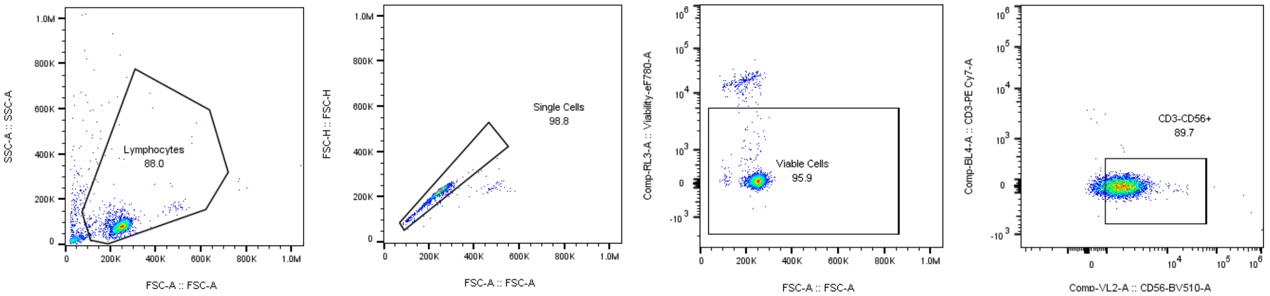

6g - CD3-CD56+^{dim} / Viability

IK14004 1.25 µM

Vehicle

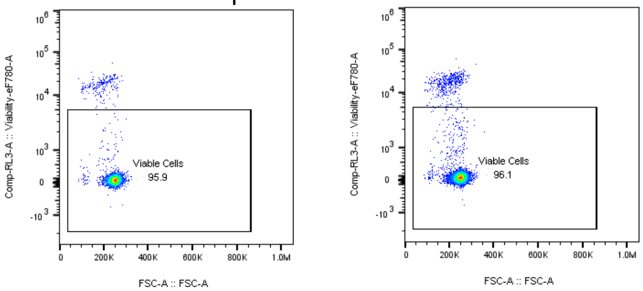

6h - CD3-CD56+^{dim} / Intracellular IL-2

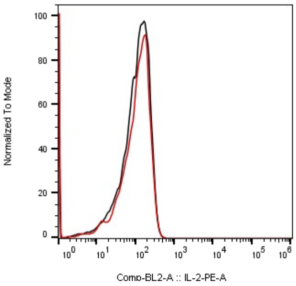

Black = vehicle
Red = IK14004 1.25 µM

The Figure shows an exemplar gating strategy from Donor 1 unstimulated NK cells (IK14004 1.25 µM). Cells gated to remove debris, doublet cell exclusion, viable cells gated and CD3-CD56+^{dim} NK cells selected for analysis of intracellular IL-2.

Figure S16: Refers to manuscript **Figure 6i and 6j**
CD25 and IL-12Rβ2 (Isolated CD3-CD56+^{dim} NK cells)

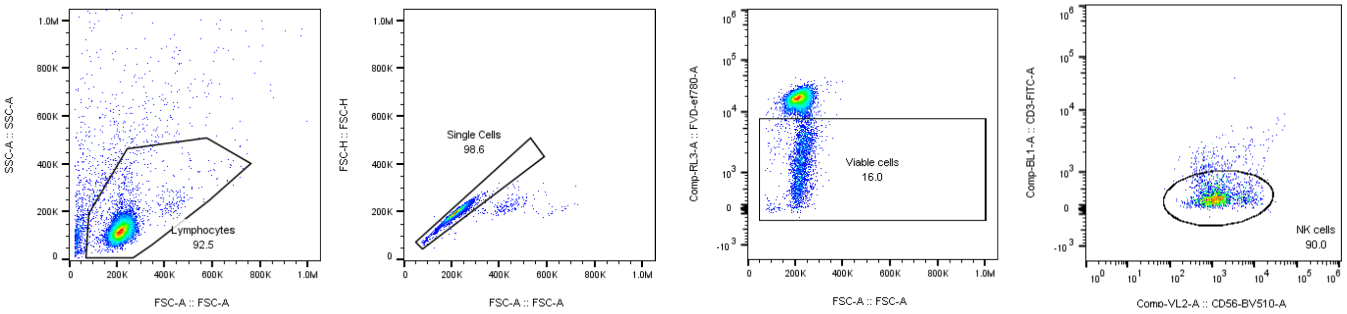

6i - CD3-CD56+^{dim} / CD25

IK14004 1.25 µM
Vehicle

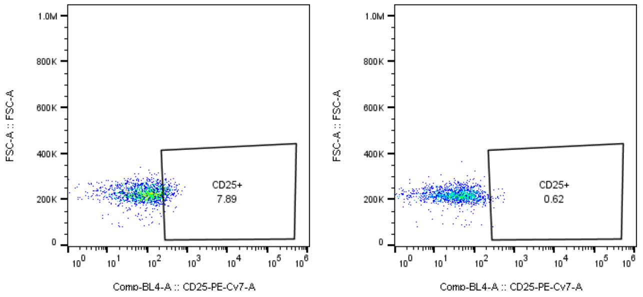

6j - CD3-CD56+^{dim} / IL-12Rβ2

IK14004 1.25 µM
Vehicle

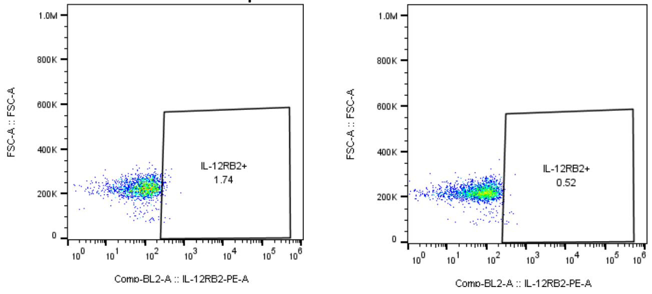

The Figure shows an exemplar gating strategy from Donor 4 unstimulated NK cells (IK14004 1.25 µM). Cells gated to remove debris, doublet cell exclusion, viable cells gated and CD3-CD56+^{dim} NK cells selected for analysis of CD25 and IL-12Rβ2.

Figure S17: Refers to manuscript **Figure 6n - 6p**

NKG2D and NKp44 (Isolated CD3-CD56+^{dim} NK cells)

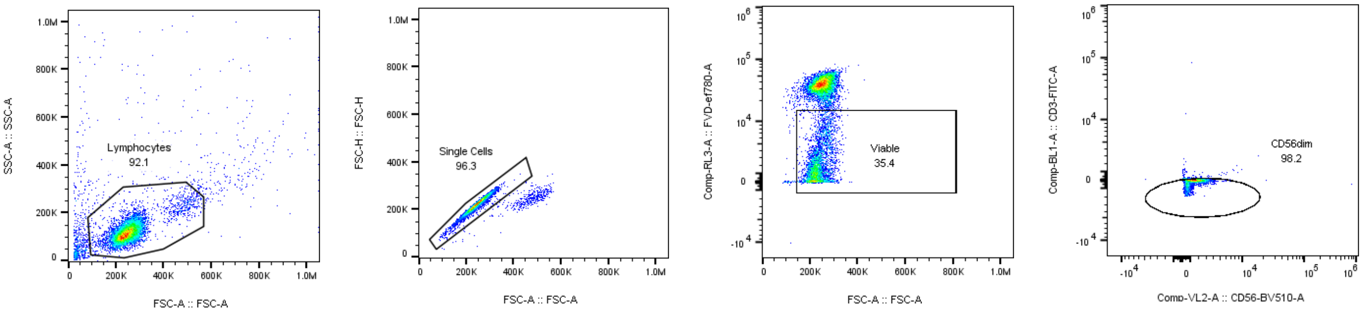

6n - CD3-CD56+^{dim} / NKG2D

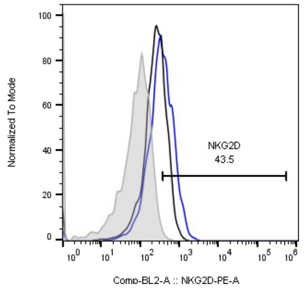

Grey = isotype
Black = vehicle
Blue = rIL-2

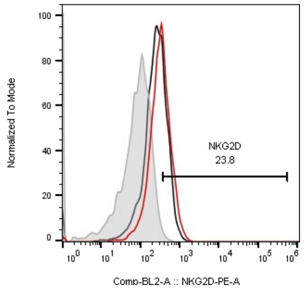

Grey = isotype
Black = vehicle
Red = IK14004 1.25 µM

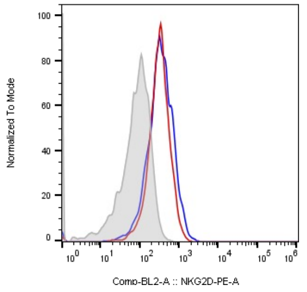

Grey = isotype
Blue = rIL-2
Red = IK14004 1.25 µM

6o - CD3-CD56+^{dim} / NKp44

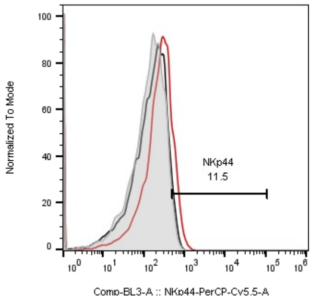

Grey = isotype
Black = vehicle
Red = IK14004 1.25 µM

6p - CD3-CD56+^{dim} / NKp44

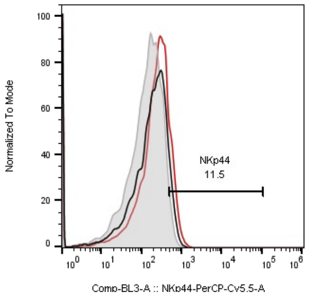

Grey = isotype
Black = rIL-2
Red = IK14004 1.25 µM

The Figure shows an exemplar gating strategy from Donor 3 unstimulated NK cells (IK14004 1.25 µM). Cells gated to remove debris, doublet cell exclusion, viable cells gated and CD3-CD56+^{dim} NK cells selected for analysis of NKG2D and NKp44.
